# Integrating Bone‐Brain Axis Modulation and Tea Consumption for Enhancing Neurovascular Resilience and Patient Rehabilitation Education

**DOI:** 10.1002/fsn3.72015

**Published:** 2026-06-26

**Authors:** Yazhen Zhang, Yisheng Chen, Zhaoyuan Huang, Shizhong Zheng, Chengwan Shen, Qiangqiang Wang, Hua Chen, Shiwei He, Qing Yang, Zemin Ou, Zijin Sun, Yuzhen Xu, Guanghui Wu, Lei Huang, John H. Zhang, Zui Zou, Wangzheqi Zhang, Shaocong Zhao

**Affiliations:** ^1^ Ningde Normal University Ningde China; ^2^ Fujian Key Laboratory of Toxicant and Drug Toxicology Medical College, Ningde Normal University Ningde China; ^3^ Department of Vascular and Interventional Radiology Ningde Municipal Hospital of Ningde Normal University Ningde China; ^4^ Ningde Clinical Medical College of Fujian Medical University Ningde Fujian Province China; ^5^ Fujian Key Laboratory of Medical Bioinformatics Fujian Medical University Fuzhou China; ^6^ Department of Neurosurgery Ningde Clinical Medical College of Fujian Medical University Ningde Fujian Province China; ^7^ Tongji Medical College Huazhong University of Science and Technology Wuhan China; ^8^ Faculty of Science Universiti Malaya Kuala Lumpur Malaysia; ^9^ Institute of Population Medicine, School of Public Health Fujian Medical University Fuzhou Fujian China; ^10^ Vegetable Science, College of Horticulture Gansu Agricultural University Lanzhou China; ^11^ China Academy of Chinese Medical Sciences Institute of Chinese Materia Medica Beijing China; ^12^ Beijing University of Chinese Medicine Beijing China; ^13^ Department of Rehabilitation The Second Affiliated Hospital of Shandong First Medical University Taian Shandong Province China; ^14^ Department of Molecular Cell and Cancer Biology University of Massachusetts Medical School Worcester Massachusetts USA; ^15^ Department of Neurosurgery, Department of Physiology and Pharmacology, Department of Neurosurgery and Anesthesiology School of Medicine, Loma Linda University Loma Linda California USA; ^16^ School of Anesthesiology Naval Medical University Shanghai China; ^17^ Xiamen University of Technology Xiamen China

**Keywords:** exercise‐induced metabolites, gut–brain axis, intracerebral hemorrhage, neurovascular protection, tea polyphenols

## Abstract

Intracerebral hemorrhage (ICH) is one of the most devastating subtypes of stroke, with limited preventive options and a challenging recovery process. This study presents a translational framework that integrates tea polyphenols (TPPs) and exercise‐induced metabolites as dual modulators of neurovascular stability, with a focus on patient education for enhancing post‐stroke recovery. By synthesizing preclinical and clinical evidence, we demonstrate how TPPs, particularly epigallocatechin gallate (EGCG), and key exercise metabolites such as lactate, β‐hydroxybutyrate, and short‐chain fatty acids (SCFAs) interact with shared redox‐sensitive and inflammatory signaling pathways (Nrf2/NF‐κB/AMPK axis) to reinforce endothelial integrity, preserve blood–brain barrier function, and maintain cerebral perfusion. These interventions also reshape the gut microbiota, promoting an SCFA‐enriched, anti‐inflammatory profile that fosters bidirectional gut‐brain communication, further stabilizing vascular homeostasis. Multi‐omics evidence suggests that TPPs and exercise metabolites may jointly regulate metabolic and immune pathways, enhancing resilience against oxidative and inflammatory injuries in the vasculature. We propose a mechanistic model in which TPPs and exercise‐derived metabolites synergistically support neurovascular function and reduce neurovascular vulnerability associated with ICH, while promoting cognitive recovery and metabolic health. Incorporating these findings into patient rehabilitation education may help individuals make informed decisions about lifestyle changes that enhance vascular health. Future research should explore the dose–response relationship, the optimal timing between tea and exercise, and individual variations, using metabolomic, microbiomic, and imaging biomarkers to personalize cerebrovascular prevention strategies.

## Graphical abstract

This figure illustrates how tea consumption and exercise metabolites may synergistically support neurovascular protection related to hemorrhagic risk. (A) Pathological mechanisms such as vascular injury, small‐vessel fragility, and impaired cerebral perfusion increase cerebrovascular vulnerability. (B) Tea polyphenols, theanine, and caffeine enhance neuroprotection, antioxidant defense, and anti‐inflammatory effects, improving vascular stability. (C) Exercise‐related metabolic adaptations support vascular repair, blood pressure regulation, and cerebral circulation. (D) Combined, tea and exercise regulate oxidative stress, modulate the gut–brain axis, and stabilize vascular function, potentially reducing hemorrhagic vulnerability and promoting cardiovascular and cognitive health.

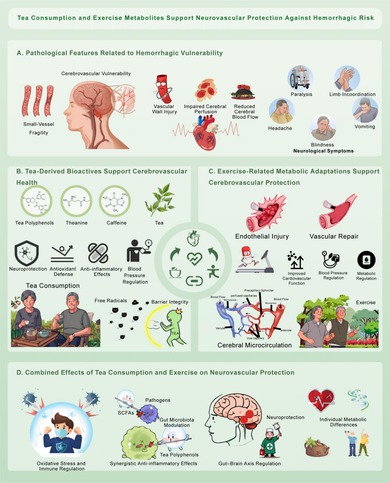

## 1 Introduction

**FIGURE 1:**
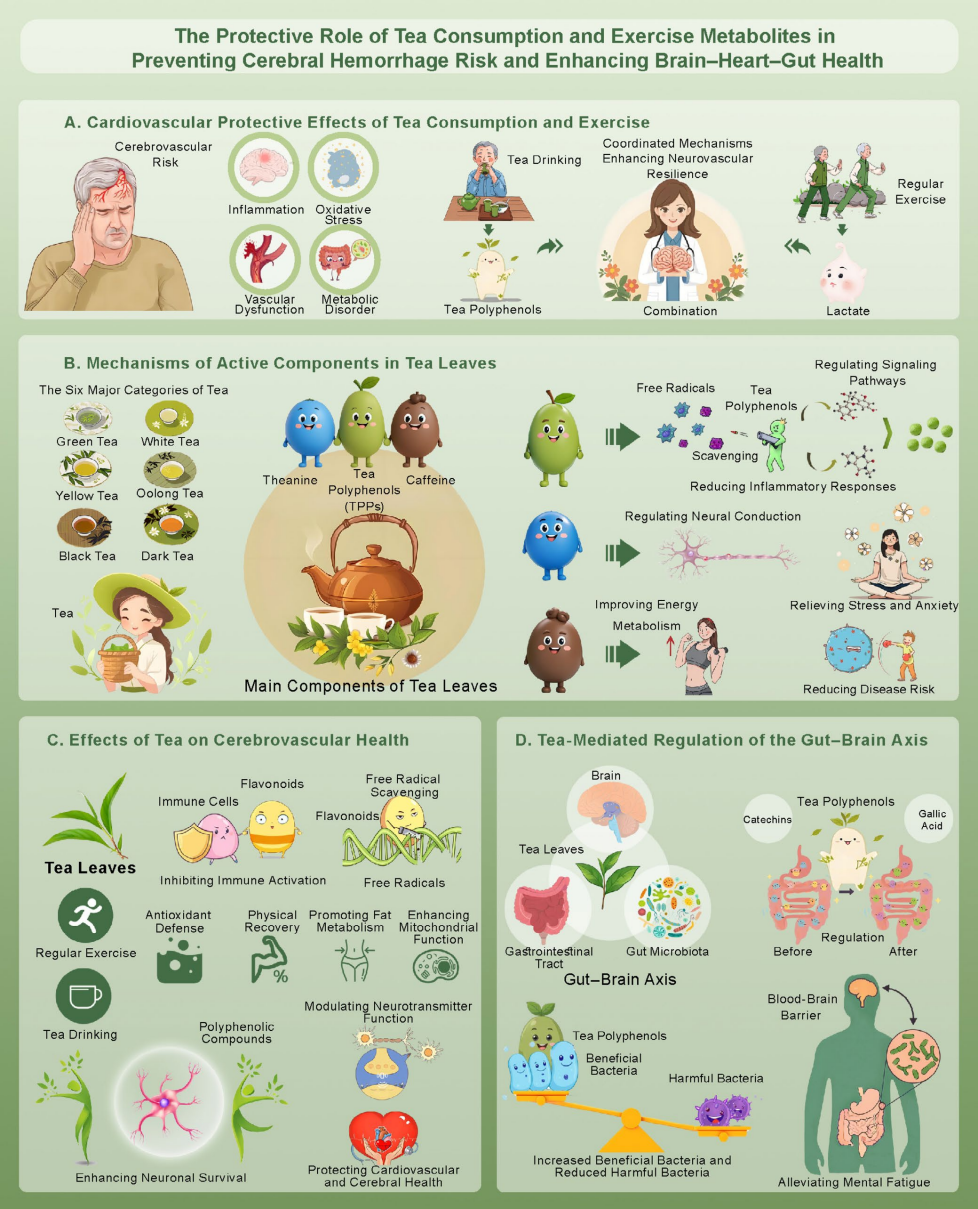
The protective role of tea consumption and exercise metabolites in preventing cerebral hemorrhage risk and enhancing brain‐heart‐gut health. (A) Cardiovascular protective effects of tea consumption and exercise: This section highlights the combined impact of tea consumption and regular exercise on reducing the risk of cerebral hemorrhage. The figure shows how tea consumption and exercise help mitigate oxidative stress, inflammation, vascular wall fragility, and metabolic disorders, which are risk factors for stroke. A combination of tea polyphenols and lactate from exercise is shown as beneficial in preventing cerebral hemorrhage. (B) The mechanisms of action of active components in tea leaves: This part explains the role of the major categories of tea (green, white, yellow, oolong, black, and dark tea) and their active components, including theanine, caffeine, and polyphenols. It outlines how these components work together to reduce free radicals, regulate neural conduction, relieve stress and anxiety, and help reduce disease risk through anti‐inflammatory pathways and improvements in energy metabolism. (C) The impact of tea on cerebral vascular health: This section illustrates the protective effects of tea on cerebral vascular health, specifically through its polyphenolic compounds. Regular tea consumption, combined with exercise, enhances immune cell function, reduces the generation of free radicals, and promotes healthy cerebrovascular function. This section emphasizes the direct effects of tea polyphenols on neurotransmitter regulation, cardiovascular health, and overall health. (D) The regulation of the gut‐brain axis by tea: The final part of the figure presents the interaction between tea and the gut‐brain axis. It shows how tea polyphenols, catechins, and gallic acid regulate gastrointestinal function, gut microbiota, and the blood–brain barrier. Tea enhances the abundance of beneficial bacteria while inhibiting harmful bacterial growth, contributing to the alleviation of mental fatigue and improved brain health.

Tea, the world's second most consumed beverage, is enriched with diverse bioactive compounds that contribute to the prevention of obesity, diabetes, hypertension, and other chronic diseases (Das et al. 2022). A growing body of experimental and clinical studies suggests that tea extracts exert protective effects on metabolic disorders, primarily through antioxidative and anti‐inflammatory mechanisms mediated by Nrf2/NF‐κB signaling (Talebi et al. 2021). Different tea varieties, including green, oolong, and black tea, exhibit distinct fermentation levels and chemical profiles, resulting in differential health benefits (Tang et al. 2019). Beyond the independent benefits of tea or physical activity alone, increasing attention has been directed toward the synergistic effects between tea polyphenols (TPPs) and exercise, which represent a promising and integrated lifestyle‐based strategy for cerebrovascular protection (Cheng et al. 2026). Recent studies suggest that this combined intervention may exert greater protective effects than either modality alone by coordinately regulating redox homeostasis, modulating inflammatory responses, improving gut–brain communication, and preserving vascular integrity, thereby enhancing neurovascular resilience (Figure 1). Importantly, TPPs and exercise metabolites provide complementary and interactive vascular benefits, and their integration offers synergistic protection against cerebrovascular damage (Figure 1; Table 1) (Nobari et al. 2021; Rojano‐Ortega 2021).

**TABLE 1:** Multidimensional cerebrovascular protective effects of tea bioactive components.

Component	Functional dimension	Core mechanism	Target/pathway	Cerebrovascular protective effects
EGCG	Antioxidant and anti‐inflammatory effects	Scavenges ROS, inhibits NF‐κB, and activates Nrf2/ARE	Nrf2/ARE and TLR4/MyD88/NF‐κB signaling	Reduces oxidative stress, preserves BBB integrity
	Cerebrovascular Hemodynamics	Promotes vasodilation, enhances endothelial NO bioavailability	eNOS/NO; AMPK/SIRT1	Improves cerebral blood flow, reduces vascular resistance
L‐Theanine	Neuroprotection	Balances glutamate/GABA, suppresses microglial activation	AMPK/mTOR, TLR4/NF‐κB	Alleviates neuroinflammation and may support cognitive function
Caffeine	Vascular Modulation & Endothelial Support	Modulates adenosine receptor signaling and endothelial‐related pathways	Adenosine receptor, eNOS/NO	May support vasodilation and reduce platelet‐related vascular risk
Pu‐erh tea‐derived polysaccharides	Gut–Brain Axis Regulation	Promotes SCFA production and modulates the gut microbiota	GPR41/GPR43; HDAC inhibition	Reduces systemic inflammation, strengthens BBB integrity
TFs	Antioxidant & Endothelial Protection	Inhibits lipid peroxidation, balances cytokine release	NF‐κB signaling; IL‐6 and TNF‐α	Attenuates cerebrovascular endothelial damage
KMP	Metabolic Modulation	Restores gut microbiota, inhibits TLR4/NF‐κB	TLR4/MyD88, PPARγ	Improves metabolic homeostasis and reduces vascular inflammatory injury
TPS	Gut Barrier & Neurovascular Protection	Upregulates tight junction proteins, regulates oxidative stress	ZO‐1/claudin‐1, TLR4/NF‐κB	Repairs intestinal barrier, mitigates endotoxemia‐related injury

*Note:* The table summarizes major tea bioactive components, highlighting their multidimensional functional roles, underlying molecular mechanisms, and key signaling pathways involved in cerebrovascular protection and brain vascular health.

Abbreviations: AMPK, AMP‐activated protein kinase; ARE, antioxidant response element; BBB, blood–brain barrier; EGCG, epigallocatechin gallate; eNOS, endothelial nitric oxide synthase; GPR41/43, G protein‐coupled receptors 41 and 43; HDAC, histone deacetylase; HIF‐1α, hypoxia‐inducible factor‐1 alpha; KMP, kaempferol; mTOR, mechanistic target of rapamycin; MyD88, myeloid differentiation primary response 88; NF‐κB, nuclear factor kappa‐light‐chain‐enhancer of activated B cells; NO, nitric oxide; Nrf2, nuclear factor erythroid 2‐related factor 2; PI3K/Akt, phosphoinositide 3‐kinase/protein kinase B; ROS, reactive oxygen species; SCFAs, short‐chain fatty acids; SIRT1, sirtuin 1; TFs, theaflavins; TLR4, toll‐like receptor 4; TPS, tea polysaccharides; ZO‐1, zonula occludens‐1.

Among tea constituents, epigallocatechin gallate (EGCG) is the most representative polyphenol with potent antioxidative and vascular‐protective properties (Li, Fang, et al. 2025). Emerging evidence suggests that EGCG may act in concert with exercise‐induced metabolic adaptations, thereby reinforcing cerebrovascular resilience through amplified antioxidant and endothelial responses (Sugita et al. 2016). Preclinical studies demonstrate that the combination of green tea and moderate‐intensity aerobic exercise improves vasodilation and vascular elasticity, thereby potentially reducing cerebrovascular vulnerability (Schimidt et al. 2014). For instance, aerobic interval training increased cerebral vasodilation by 29%, indicating a dose‐dependent relationship between exercise intensity and vascular benefits (Moir et al. 2024). Furthermore, EGCG can also work in conjunction with other plant‐derived bioactive compounds through a multi‐targeted synergy mechanism, thereby jointly enhancing vascular protection (Xu et al. 2024). In parallel, TPPs directly enhance endothelial function and reduce platelet aggregation, while exercise optimizes hemodynamics and shear stress, together conferring comprehensive cerebrovascular protection. Furthermore, lifestyle interventions that integrate green tea intake with healthy dietary habits and structured exercise have been shown to lower the incidence of cardiovascular disease and diabetes. Green tea, which is rich in EGCG, is generally considered safe and well tolerated, with long‐term daily intake equivalent to 8–16 cups of tea reported to be well tolerated in humans (Hu et al. 2018). At the molecular level, the health‐promoting effects of polyphenols are largely attributed to their antioxidant and anti‐inflammatory properties. Polyphenols not only directly scavenge free radicals but also enhance endogenous antioxidant defense systems. They are broadly classified into flavonoids and non‐flavonoids, with flavonoids being the most extensively studied for their antioxidative, immunomodulatory, and circulatory effects (Lee et al. 2015). Notably, their bioavailability is strongly influenced by gut microbiota composition, highlighting inter‐individual variability in therapeutic outcomes.

Recent studies further demonstrate that polyphenol supplementation provides significant health benefits in athletes by optimizing recovery and performance. Antioxidants help maintain immune function by lowering reactive oxygen species (ROS) levels and modulating calcium signaling and redox reactions in skeletal muscle fibers during exercise. Building on these findings, the combined application of green TPPs and exercise has been proposed as an innovative non‐pharmacological model that leverages their synergistic molecular and physiological interactions (Peternelj and Coombes 2011). Importantly, accumulating evidence indicates that TPPs and exercise metabolites converge on the Nrf2–NF‐κB pathway to jointly enhance cerebrovascular health (Bentley et al. 2014). Taken together, existing studies provide a plausible mechanistic framework for the synergistic neurovascular protective effects of TPPs and exercise, supporting their potential role in cerebrovascular protection. Importantly, while metabolic disorders such as obesity, hypertension, and diabetes are discussed throughout this review, they are considered primarily as upstream contributors to cerebral small vessel dysfunction and BBB vulnerability. Accordingly, the central focus of this review is on intracerebral hemorrhage (ICH), cerebral small vessel disease, and neurovascular integrity, with metabolic and systemic factors incorporated mainly to contextualize mechanistic pathways relevant to hemorrhagic risk and vascular fragility.

## 2 Protective Effects of Tea

### 2.1 Active Compounds in Tea

Green tea, widely consumed especially in Eastern countries, exerts multiple health benefits largely attributed to its catechins, particularly EGCG (Reygaert 2017). Together with other bioactive constituents such as caffeine and amino acids, these compounds enhance antioxidant defenses, support exercise recovery, and improve vascular health (Nobari et al. 2021; Braschi et al. 2023). EGCG, the predominant catechin in green tea, demonstrates potent metabolic and vascular protective effects. These include enhanced fat oxidation, reduced cholesterol, and improved antioxidant and anti‐inflammatory responses (Quan et al. 2025). Other tea‐derived bioactive compounds also contribute to gut microbiota modulation and systemic health (Suzuki et al. 2016). TPPs attenuate inflammation by suppressing myeloperoxidase (MPO) in neutrophils, thereby protecting the intestinal barrier and regulating immune responses. Network pharmacology suggests that the PI3K/Akt/mTOR targets of TPPs overlap with exercise metabolites, indicating convergent regulation of cerebral blood flow. In contrast, tea polysaccharides (TPS) and theaflavins (TFs) exert weaker effects on MPO and cytokine inhibition, while tea anthocyanins (TA) display only modest anti‐inflammatory activity (Xu et al. 2023). Collectively, these bioactive components suppress pro‐inflammatory mediators such as COX2, IL‐1β, TNF‐α, and iNOS by activating Nrf2/ARE and inhibiting TLR4/MyD88/NF‐κB signaling, thereby reinforcing intestinal antioxidant and anti‐inflammatory defenses. The active compounds in tea also play a critical role in regulating obesity and metabolic syndrome. Green tea extracts modulate gut microbiota in high‐fat diet (HFD)‐fed mice, alleviating metabolic disorders and preventing obesity (Hodgson et al. 2013). For example, green TPPs improve glucose tolerance, reduce fatty liver, and enhance the abundance of beneficial gut bacteria. They also mitigate HFD‐induced intestinal inflammation and barrier dysfunction, which has systemic metabolic implications (Suzuki et al. 2012). Green tea catechins additionally display broad antioxidant properties. Mechanistically, they induce antioxidant enzymes such as superoxide dismutase (SOD), catalase (CAT), and glutathione peroxidase (GPx), scavenge ROS, and inhibit lipid peroxidation. Previous systematic reviews have reported that green tea supplementation enhances total antioxidant capacity and dose‐dependently reduces lipid peroxidation markers such as malondialdehyde (MDA). While some studies found no significant effect on total antioxidant capacity and MDA in type 2 diabetes patients, reductions in C‐reactive protein (CRP) suggest potential regulation of systemic inflammation. Moreover, findings from published meta‐analyses suggest significant associations between green tea supplementation and reductions in body weight and body mass index (BMI), supporting its role in weight management and functional food development.

#### 2.1.1 Tea Polyphenols

TPPs, including catechins, flavonoids, anthocyanins, and phenolic acids, exert multifaceted antioxidative and anti‐inflammatory effects (Feng et al. 2024). EGCG, the most abundant catechin, scavenges ROS, reduces lipid peroxidation, and activates the Nrf2/ARE pathway (Xu et al. 2024). This induces antioxidant enzymes such as SOD, CAT, HO‐1, and NQO‐1, while suppressing inflammatory mediators including IL‐1β, TNF‐α, and COX‐2 (Mao et al. 2019). Flavonoids like quercetin and kaempferol inhibit NF‐κB signaling, reducing cytokine release, strengthening intestinal barrier integrity, and alleviating inflammation, including exercise‐induced intestinal stress (Long et al. 2023). TPPs also preserve mitochondrial function by reversing ROS‐induced damage, stabilizing membranes, upregulating Bcl‐2, and downregulating Cytc/caspase‐3 signaling, thereby supporting neuronal survival and immune regulation (Jia et al. 2021). In the nervous system, polyphenols enhance synaptic plasticity and cognition via BDNF and CREB signaling, providing neuroprotection after cerebrovascular injury (Rebas et al. 2020; Campos‐Esparza and Torres‐Ramos 2010). In addition, they regulate bile acid metabolism and reshape gut microbiota composition, thereby supporting the gut–brain axis and reducing intestinal inflammation (Wu et al. 2024). Different tea varieties display distinct polyphenol profiles. Green tea extract (GTE), rich in EGCG, scavenges ROS and inhibits lipid peroxidation, enhancing antioxidant status in metabolic syndrome and obesity. In contrast, steeped tea extracts (STE) and Pu‐erh tea contain anthocyanins, flavonoids, tannins, and TFs, which act synergistically to reduce oxidative stress and strengthen endogenous antioxidant defenses (Chen et al. 2024; Zhao et al. 2025). Clinical studies further demonstrate that catechins promote fat oxidation, prevent hepatic steatosis, improve insulin sensitivity, and alleviate NAFLD. Moreover, EGCG supplementation reduces inflammation and fibrosis, lowering the risk of hepatocellular carcinoma (Sojoodi et al. 2020). In summary, TPPs, particularly EGCG, exert comprehensive protective effects by modulating oxidative stress, inhibiting NF‐κB signaling, preserving mitochondrial and endothelial function, and regulating the gut–brain axis. These mechanisms provide a strong molecular basis for potential applications in the prevention and management of cerebrovascular and metabolic diseases.

#### 2.1.2 Theanine and Caffeine

L‐theanine, a non‐protein amino acid abundant in tea, exerts neuroprotective, anti‐inflammatory, and gut‐barrier regulatory effects. It reduces IL‐6 and NF‐κB activation in LPS‐induced models while maintaining glutamate‐GABA balance, thereby preventing excitotoxicity (Chandrasekhar et al. 2017). In addition, L‐theanine strengthens intestinal barrier integrity by up‐regulating tight junction proteins such as ZO‐1 and claudin‐1 and lowering circulating LPS, mitigating systemic inflammation (Wang et al. 2024). Pu‐erh tea, rich in theanine derivatives, further protects the blood–brain barrier and suppresses hippocampal TLR4/MyD88/NF‐κB signaling, alleviating stress‐induced neuroinflammation (Jeong et al. 2020). Caffeine, another major tea component, enhances energy metabolism, promotes lipolysis, and supports vascular function. In high‐fat diet–induced NASH models, caffeine combined with EGCG improves hepatic lipid accumulation, inflammation, and fibrosis. Mechanistically, caffeine elevates adipose tissue cAMP, stimulating triglyceride hydrolysis and reducing fat storage. It also limits endothelial‐to‐osteoblast transition and immune cell proliferation, thereby preserving vascular integrity (Kakuda 2011). When used together, L‐theanine and caffeine impart beneficial effects. While the former inhibits neurotransmission and promotes gut barrier function, the latter enhances lipid metabolism and vascular function. Collectively, they enhance tea's broad protective effects against neuroinflammation, metabolic disorders and vascular dysfunction (Higdon and Frei 2003).

#### 2.1.3 Other Bioactive Compounds

Flavonoids such as kaempferol (KMP) represent another important class of tea‐derived bioactive compounds. KMP exhibits antioxidant, anti‐inflammatory, and antimicrobial properties, and preclinical studies show it improves metabolic health by reducing body and liver weight, lowering glucose and cholesterol, and restoring gut microbiota diversity in HFD‐fed mice (Wang et al. 2020). KMP also reinforces intestinal barrier integrity and suppresses TLR4/NF‐κB signaling, while increasing SCFA‐producing or microbiota‐associated bacteria such as *Butyrivibrio* (Periferakis et al. 2022). TPS, another significant group of bioactive compounds, reach the colon largely intact and are metabolized into SCFAs. These metabolites regulate gut homeostasis and systemic metabolism. Flavonoids and TPS derivatives modulate lipid metabolism via hormone‐sensitive lipase, acetyl‐CoA carboxylase, carnitine acyltransferase, and peroxisome proliferator‐activated receptors (PPARs), enhancing lipid utilization and energy expenditure (Mulvihill and Huff 2012). Experimental studies confirm that anthocyanins, catechins, and TPS improve gut barrier function, reduce oxidative stress, and alleviate inflammation in models of HFD‐induced or exercise‐induced barrier disruption (Kung et al. 2020, 2022). Other tea compounds, including theabrownins (TB) and YLGT from Pu‐erh tea, also support gut health by reducing fat accumulation and improving intestinal pathology (Tian et al. 2024). Together, flavonoids, polysaccharides, and other tea‐derived compounds act through antioxidative, anti‐inflammatory, and microbiota‐modulating pathways to enhance gut barrier integrity, reduce low‐grade inflammation, and support systemic metabolic and neurological health (Dong et al. 2025).

### 2.2 Effects of Tea on Cerebrovascular Health

Tea flavonoids confer cardiovascular and neuroprotective benefits beyond their antioxidant and anti‐inflammatory activities. In vitro and animal studies suggest potential improvements in vascular function and cognition, as well as attenuation of metabolic syndrome, allergies, asthma, and IBD. Clinical data indicate that quercetin supplementation reduces systolic blood pressure and inflammatory markers in patients with metabolic syndrome, though optimal doses require validation in randomized controlled trials (Debnath et al. 2025).

#### 2.2.1 Tea and Improvement of Cerebrovascular Hemodynamics

**FIGURE 2:**
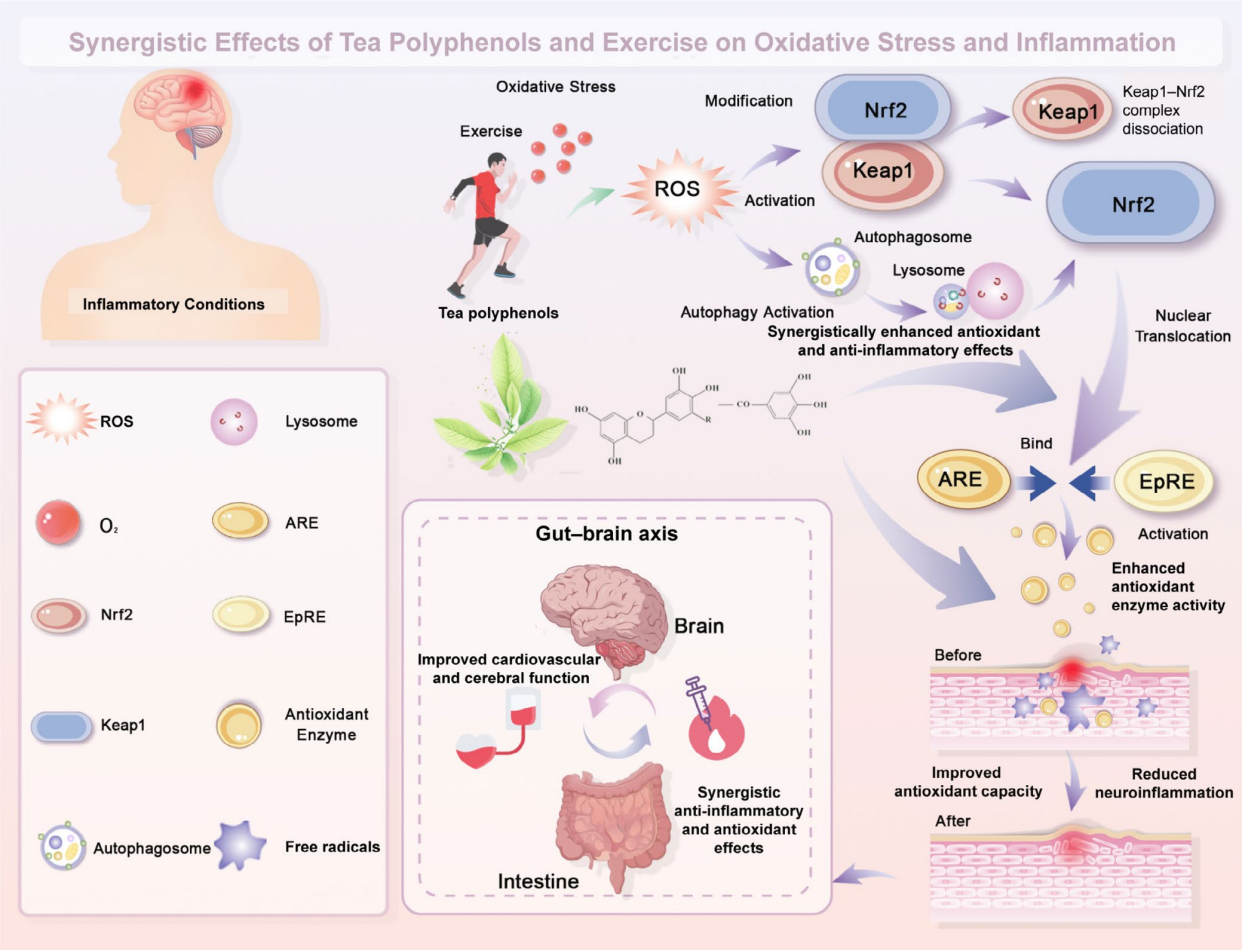
Synergistic effects of tea polyphenols and exercise on oxidative stress, antioxidant capacity, and inflammatory responses. This figure illustrates the synergistic effects of tea polyphenols and exercise in enhancing antioxidant capacity and reducing inflammation, particularly under inflammatory conditions. Exercise‐induced reactive oxygen species (ROS) activate the Nrf2‐Keap1 signaling pathway, leading to the upregulation of antioxidant genes and the enhancement of antioxidant enzyme activity. Tea polyphenols may further strengthen these effects and promote autophagy, during which autophagosomes fuse with lysosomes to remove cellular waste and reduce oxidative damage. The figure also highlights the influence of this combination on the brain‐gut axis, thereby improving both cardiovascular and cerebral function while exerting anti‐inflammatory and antioxidant effects in the brain and intestine. The before‐and‐after comparison shows reduced inflammatory markers, enhanced antioxidant capacity, and attenuated inflammation, emphasizing the beneficial effects of the combined intervention of tea and exercise.

TPPs act synergistically with exercise‐induced metabolic responses to enhance cardiovascular and cerebrovascular resilience (Table 1; Figure 2) (Ghosh et al. 2012). Habitual tea consumption, particularly of flavan‐3‐ols and flavonols, is consistently associated with reduced incidence of and mortality from atherosclerotic cardiovascular diseases. Benefits are most pronounced at 2–4 cups daily, while higher intake correlates with reduced digestive disease mortality. These dose‐dependent effects underscore tea's protective role in cardiovascular outcomes.

#### 2.2.2 Tea's Neurovascular Protective Effects and Vascular Repair

TPPs enhance neuronal survival by preserving mitochondrial function, reducing neuroinflammation, and scavenging ROS (Figure 2) (Sandoval‐Acuña et al. 2014). They cross the blood–brain barrier, modulate neurotrophic factors such as BDNF, and improve cognition (Qi et al. 2017). Advanced delivery systems, including lipid nanoparticles, further improve their bioavailability. In addition, polyphenols regulate neurotransmitters such as dopamine, GABA, and glutamate, mitigating neuroinflammation‐related deficits and potentially contributing to neurovascular protection (Nabavi et al. 2014). In Alzheimer's disease and other protein misfolding disorders, polyphenols inhibit toxic fibril formation, promote non‐toxic aggregates, and reduce membrane toxicity, inflammation, and apoptosis. Quercetin protects diabetic cerebrovascular endothelial cells via VCAM1 modulation, while tea‐derived bioactive compounds may support endothelial repair and vascular homeostasis (Huang et al. 2022).

#### 2.2.3 Tea's Regulation of the Gut‐Brain Axis

The human gut microbiota, dominated by *Bacteroidetes* and *Firmicutes*, plays a central role in metabolic and immune homeostasis (Yang et al. 2022). Dysbiosis promotes inflammation and contributes to systemic and neurological disorders, whereas probiotics such as *Lactobacillus* and *Bifidobacterium* generate SCFAs, enhance barrier integrity, and modulate oxidative stress (Westfall et al. 2017). Bidirectional communication between the gut and brain occurs through immune, endocrine, and metabolic pathways. Microbial metabolites, including SCFAs and neurotransmitter‐related compounds, as well as host factors such as brain‐derived neurotrophic factor (BDNF), play a crucial role in regulating cognition and behavior (Liu et al. 2023). For example, butyrate has been shown to enhance BDNF expression and facilitate memory consolidation, whereas dysbiosis has been associated with various mood and cognitive disorders. TPPs such as epicatechin (EC) and EGCG modulate gut microbiota by suppressing pathogens and enriching beneficial species including *Lactobacillus*, *Bifidobacterium*, and *Akkermansia*. These interactions enhance SCFA production, strengthen barrier integrity, and facilitate gut–brain communication, thereby exerting antioxidant, anti‐inflammatory, and neuroprotective effects. EGCG also mitigates brain aging via antioxidant enzyme activation and HPA‐axis modulation, while its metabolites, including acetate, propionate, and butyrate, further support energy metabolism and immune regulation (Luo et al. 2022; Pervin et al. 2019). In addition, polyphenols regulate neurotransmitter balance and fatigue by enhancing energy metabolism. They also reshape gut microbiota by suppressing harmful species and promoting polyphenol‐metabolizing bacteria, which in turn strengthens gut barrier function and cerebrovascular resilience (Truyens et al. 2024). When combined with exercise, TPPs enhance mitochondrial biogenesis in perivascular cells, increase cerebral blood flow, and promote neuroplasticity and cognition (Láng et al. 2024). SCFAs contribute to gut and vascular health by regulating immunity, reducing inflammation, and maintaining BBB integrity. TPPs and polysaccharides enhance these effects, preserving gut–brain axis function and cognitive performance (Martin et al. 2018; Petra et al. 2015). Dysbiosis compromises BBB integrity, but SCFAs and tea‐derived phytochemicals counteract this by reducing neuroinflammation (Hu et al. 2023). In summary, tea‐derived polyphenols, polysaccharides, and other bioactives exert multifaceted cerebrovascular protection by improving hemodynamics, reducing neuroinflammation, promoting vascular repair, and regulating the gut–brain axis. These mechanisms complement exercise‐derived metabolites, setting the stage for synergistic neurovascular protection discussed in the next section.

## 3 Cerebrovascular Protection by Exercise Metabolites

**FIGURE 3:**
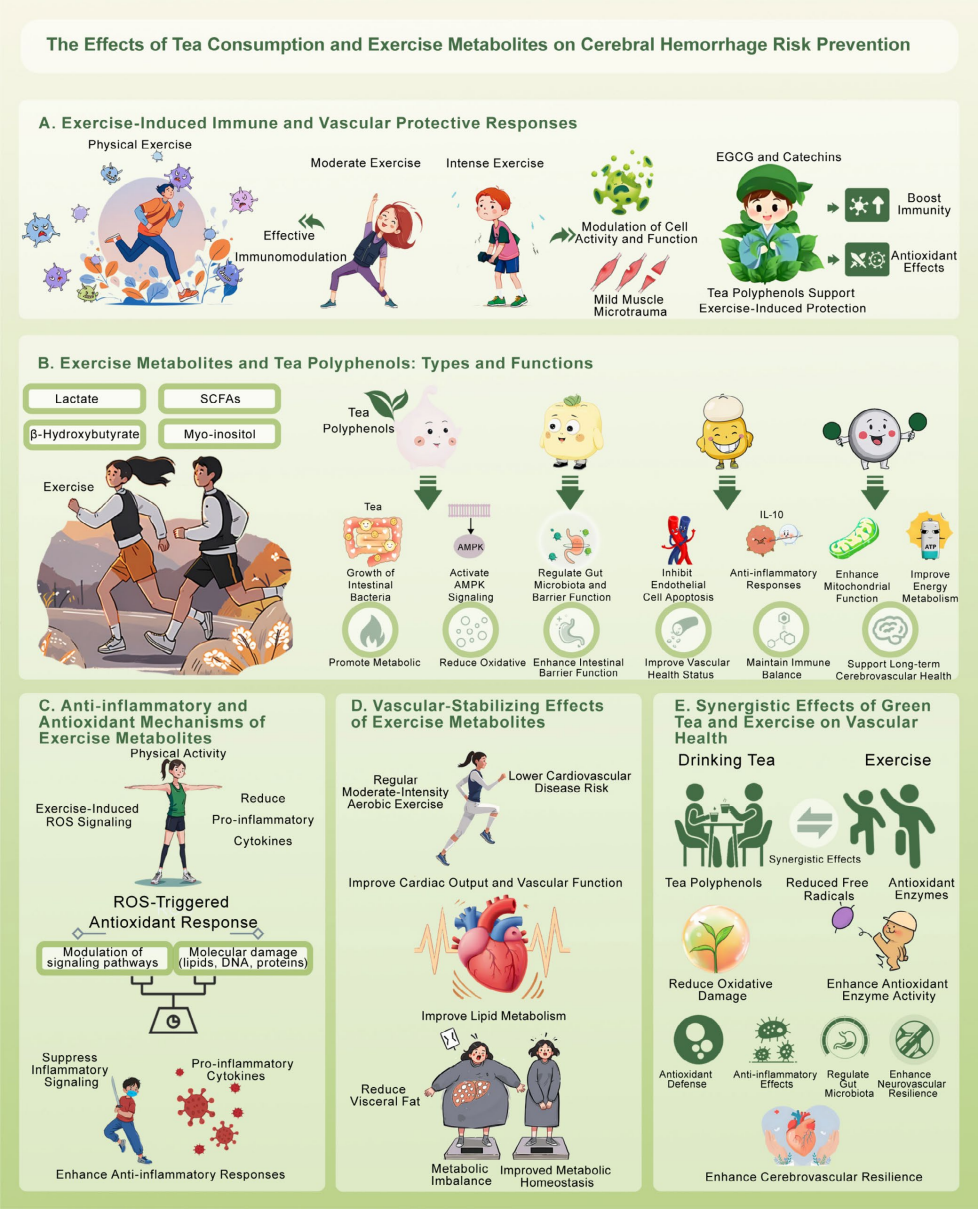
The synergistic effects of tea consumption and exercise metabolites in preventing cerebral hemorrhage risk and promoting cardiovascular health. (A) Cerebrovascular protective effects of exercise metabolites: This panel highlights the role of different exercise intensities, particularly moderate and high‐intensity exercise, in enhancing immune function and modulating cellular responses. It illustrates that moderate and high‐intensity exercise may induce mild muscle microdamage, which can trigger adaptive immune responses and cellular remodeling. Exercise‐induced metabolites, such as lactate, may further promote immune regulation and support vascular health. (B) Types and functions of exercise metabolites: This section shows the relationship between exercise metabolites, such as lactate, short‐chain fatty acids (SCFAs), β‐hydroxybutyrate, and inosine, and tea components, particularly tea polyphenols, including catechins such as epigallocatechin gallate (EGCG). Together, these factors may support the growth of beneficial gut bacteria, regulate oxidative stress, enhance metabolic function, and improve vascular health. It further illustrates how these combinations may modulate pathways such as AMPK, reduce inflammation, and attenuate endothelial cell apoptosis, thereby improving vascular function and reducing the risk of cerebrovascular injury. (C) Anti‐inflammatory and antioxidative mechanisms of exercise metabolites: This part highlights how physical exercise increases reactive oxygen species (ROS) production, thereby triggering antioxidant responses and modulating inflammatory pathways. Exercise‐induced antioxidant defenses help counteract oxidative stress and reduce the production of pro‐inflammatory cytokines, thereby limiting damage to lipids, DNA, and proteins while enhancing anti‐inflammatory responses. (D) Vascular‐stabilizing effects of exercise metabolites: Long‐term aerobic exercise is associated with a lower risk of cardiovascular disease, increased cardiac output, and improved lipid metabolism. This section also emphasizes that exercise helps reduce visceral fat and maintain metabolic homeostasis, thereby supporting vascular stability and overall cardiovascular health. (E) Synergistic effects of green tea and exercise: This section illustrates how drinking green tea in combination with exercise may enhance antioxidant and anti‐inflammatory activities. The interaction of tea polyphenols with exercise‐induced signaling and antioxidant enzymes may help reduce oxidative damage, regulate the gut microbiota, and lower the risk of cerebrovascular dysfunction, thereby contributing to improved vascular health.

Physical exercise functions as a potent immunomodulator, protecting the body from pathogens and facilitating tissue repair after injury. Skeletal muscle stress or damage triggers a delicate balance between pro‐ and anti‐inflammatory responses, which is essential for effective regeneration; prolonged or excessive inflammation, however, impairs recovery and tissue homeostasis (Dufresne et al. 2016). Nutritional and metabolite‐based strategies have therefore been proposed to enhance recovery and optimize immune regulation. During exercise, circulating leukocytes transiently increase as immune cells are mobilized from lymphoid tissues, vessel walls, and the spleen (Figure 3) (Adams et al. 2011). Moderate exercise strengthens immune function by improving antigen recognition, presentation, and effector responses, thereby reducing infection severity and shortening recovery time (Simpson et al. 2015). Conversely, prolonged or high‐intensity exercise, such as marathon running, can suppress immunity, decreasing the numbers or activity of T cells, NK cells, neutrophils, and salivary IgA, thus illustrating the dual immunomodulatory effects of exercise. Exercise‐induced muscle microtrauma provokes an inflammatory cascade that activates satellite cells and stimulates new muscle fiber formation (Bazgir et al. 2017). While critical for regeneration, excessive microtrauma or sustained inflammation limits performance and delays recovery. Consequently, approaches that attenuate inflammation or accelerate recovery through metabolites or nutrition may amplify the adaptive benefits of exercise (Chazaud 2016). Polyphenolic compounds, abundant in tea and other plant‐derived foods, have gained attention for their immunometabolic and antioxidant effects. Although their role in post‐exercise muscle recovery remains under investigation, mounting evidence suggests they interact with exercise‐induced metabolites to synergistically enhance vascular and neuroprotective functions.

### 3.1 Effects of Exercise Metabolites on Brain Vasculature

Exercise not only strengthens muscle performance but also induces systemic metabolic and immune adaptations that restore homeostasis and reinforce vascular resilience (Figure 3) (Ashcroft et al. 2024; Qiu et al. 2023). A variety of metabolites produced during physical activity, including lactate, SCFAs, β‐hydroxybutyrate (BHB), and myo‐inositol, serve dual roles as both energy substrates and signaling molecules. Collectively, these metabolites regulate oxidative stress, immune balance, mitochondrial activity, and vascular stability (Table 2) (Jang et al. 2023). Regular, moderate‐intensity exercise amplifies their production, thereby improving metabolic efficiency, enhancing antioxidant defenses, and supporting long‐term cerebrovascular health (Yang and Kwon 2020; Shi et al. 2025).

**TABLE 2:** Classification and cerebrovascular protective functions of exercise‐related metabolites and signaling mediators.

Metabolite	Core mechanism	Target/pathway	Cerebrovascular protective effects
Lactate	Activates AMPK signaling, reduces ROS, promotes endothelial repair	AMPK, NAD+/SIRT1	Improves vascular function, reduces oxidative stress
SCFAs	Inhibit inflammation via GPR41/GPR43 and enhance gut barrier function	GPR41/GPR43, HDAC inhibition	Reduces systemic inflammation, improves cerebrovascular health
β‐Hydroxybutyrate	Enhances antioxidant enzyme activity, inhibits endothelial apoptosis	SIRT1 and antioxidant enzymes, including SOD and CAT	Reduces oxidative stress and protects endothelial integrity
Inosine	Enhances mitochondrial function and ATP production	Mitochondrial respiratory chain, ATP synthase	Supports cerebrovascular energy metabolism and may reduce vascular fragility
Reactive oxygen species (ROS)	Activates Nrf2/ARE pathway, enhances antioxidant enzyme expression	Nrf2/ARE, NF‐κB	Moderate ROS triggers adaptive antioxidant responses and may reduce oxidative damage
Nitric oxide (NO)	Improves vasodilation via eNOS/NO pathway	eNOS/NO, cGMP	Enhances vascular elasticity and supports blood pressure regulation

*Note:* Key exercise‐induced metabolites, including lactate, SCFAs, β‐hydroxybutyrate, Myo‐inositol, ROS, and NO, collectively support cerebrovascular health by improving vascular function, reducing oxidative stress, maintaining endothelial integrity, and modulating inflammation.

Abbreviations: AMPK, AMP‐activated protein kinase; ATP, adenosine triphosphate; BHB, β‐hydroxybutyrate; CAT, catalase; cGMP, cyclic guanosine monophosphate; eNOS, endothelial nitric oxide synthase; GPR41/GPR43, G protein–coupled receptors 41/43; HDAC, histone deacetylase; NAD+, nicotinamide adenine dinucleotide; NF‐κB, nuclear factor kappa B; NO, nitric oxide; Nrf2/ARE, nuclear factor erythroid 2‐related factor 2/antioxidant response element; ROS, reactive oxygen species; SCFAs, short‐chain fatty acids; SIRT1, sirtuin 1; SOD, superoxide dismutase.

#### 3.1.1 Brain Vascular Protection of Lactate

**FIGURE 4:**
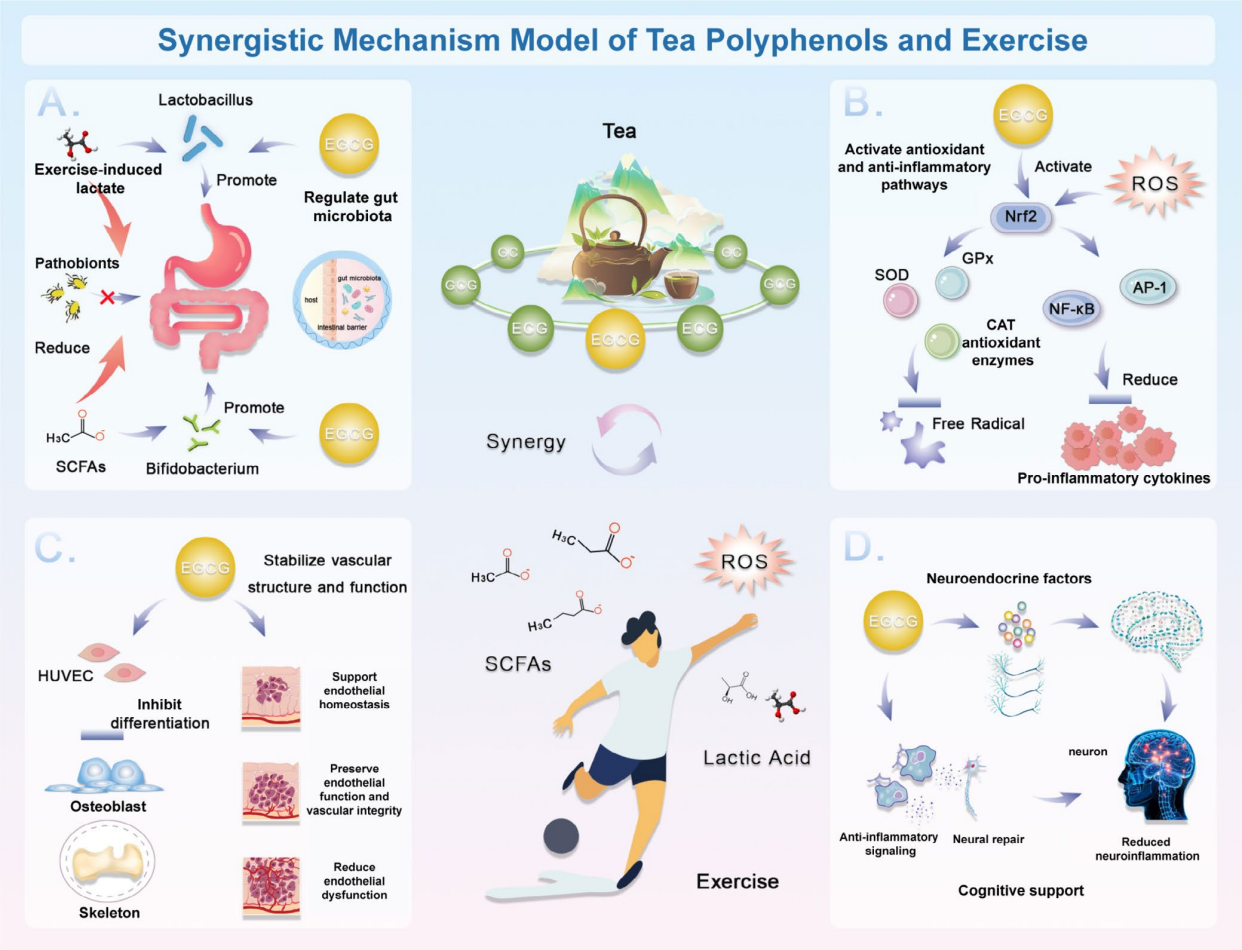
Synergistic effects of tea polyphenols and exercise on gut microbiota, antioxidant pathways, vascular function, and cognitive health. (A) Regulation of gut microbiota: Tea polyphenols promote beneficial bacteria, such as Lactobacillus and Bifidobacterium, and SCFA production. (B) Activation of antioxidant and anti‐inflammatory pathways: Tea and exercise may activate Nrf2‐related signaling, enhancing antioxidant defenses. (C) Stabilization of vascular function: SCFAs and exercise metabolites support endothelial integrity. (D) Neuroendocrine and cognitive benefits: Their synergistic effects may support neuronal repair and cognitive function.

Lactate, a hallmark product of anaerobic glycolysis, acts not only as an energy substrate but also as a signaling molecule influencing vascular and metabolic health. Lactic acid can also regulate the expression of related genes by inducing a novel epigenetic modification called lactylation, thereby influencing inflammatory responses, metabolic reprogramming, and vascular homeostasis (Tang et al. 2025). By activating the AMPK pathway, lactate alleviates oxidative stress, enhances endothelial resilience, and supports cerebrovascular protection (Huang et al. 2021). Exercise‐induced lactate also promotes the growth of SCFA‐producing gut bacteria, linking muscular activity to gut‐derived metabolic benefits and further reducing cerebrovascular risk through the gut–brain axis (Figure 4) (Huang et al. 2021). Excessive lactate accumulation during intense exercise contributes to fatigue; therefore, enhancing lactate clearance, often monitored by lactate dehydrogenase (LDH) activity, is critical for recovery (Lee et al. 2023). Experimental evidence shows that TPPs and wolfberry extract enhance LDH activity, accelerate lactate metabolism, and lower circulating lactate levels, thereby improving performance and recovery (Bi et al. 2023). Beyond these effects, lactate protects mitochondria and maintains energy balance via AMPK–SIRT1 signaling, while SCFA/GPR41/43 activation further optimizes lipid and glucose metabolism. Together, these pathways contribute to vascular resilience and muscle repair (Fang et al. 2018; Bongiovanni et al. 2021).

#### 3.1.2 Brain Vascular Protection by Short‐Chain Fatty Acids

SCFAs, primarily acetate, propionate, and butyrate, are produced by microbial fermentation of dietary polysaccharides (Kasubuchi et al. 2015). Although SCFAs are classically regarded as gut microbiota–derived metabolites, accumulating evidence indicates that physical exercise modulates SCFA production indirectly by reshaping gut microbial composition and metabolic activity, thereby linking these metabolites to exercise‐responsive metabolic pathways relevant to brain vascular health (Cullen et al. 2023). While *Lactobacillus* and *Bifidobacterium* are recognized contributors to SCFA biosynthesis in the gut, recent studies emphasize that exercise‐induced alterations in microbial diversity and substrate utilization significantly enhance circulating SCFA availability, which in turn affects systemic and cerebrovascular function. Their typical ratio is approximately 60:20:20, with propionate and butyrate exerting the strongest anti‐inflammatory and barrier‐protective effects (Liu et al. 2021). Individuals adhering to WHO dietary and activity guidelines display higher microbial diversity and greater SCFA production compared with sedentary individuals. Furthermore, exercise‐associated increases in SCFA levels have been shown to attenuate endothelial inflammation, suppress pro‐inflammatory cytokine release, and enhance cerebrovascular resilience, suggesting that SCFAs function as downstream mediators of exercise‐induced vascular protection rather than independent microbial by‐products. Acetate promotes IgA secretion through GPR43, whereas butyrate inhibits HDAC activity to enhance Foxp3+ Treg differentiation while strengthening the integrity of the epithelial barrier (Xu et al. 2018). Beyond immunity, SCFAs function as signaling molecules that modulate glucose and lipid metabolism across colon, liver, and brain tissues (Table 2). Propionate contributes to hepatic gluconeogenesis and insulin sensitivity, while butyrate supports mitochondrial metabolism and upregulates BDNF, promoting neurogenesis and memory (Yu et al. 2020; Weitkunat et al. 2016). Through these mechanisms, exercise‐modulated SCFA signaling indirectly supports cerebrovascular homeostasis and neurovascular coupling. Synergistically, TPS and polyphenols further potentiate exercise‐related increases in butyrate production, suppressing ROS and NF‐κB–mediated inflammation while reinforcing intestinal barrier function (Shen et al. 2023; Ghosh et al. 2021). Pu‐erh tea further remodels the gut microbiota, increasing acetate and propionate, thereby conferring anti‐inflammatory and neuroprotective benefits. Although SCFAs reach the brain at relatively low concentrations, their exercise‐responsive systemic actions contribute substantially to cerebrovascular protection via gut–brain axis signaling and neurotrophic support.

#### 3.1.3 Brain Vascular Protection by β‐Hydroxybutyrate

BHB, a key ketone body elevated during exercise, exerts strong cerebrovascular protective effects (Li, Qiu, et al. 2025). Unlike lactate and SCFAs, which primarily act through gut microbiota and AMPK pathways, BHB directly enhances endogenous antioxidant defenses by increasing SOD and CAT activity, inhibiting endothelial apoptosis, and maintaining vascular integrity. In the brain, BHB serves as both an energy substrate and a signaling molecule, improving mitochondrial efficiency, up‐regulating BDNF, modulating neurotransmitters such as serotonin, and attenuating neuroinflammation (Lima Giacobbo et al. 2019). These properties make BHB a potent complement to lactate and SCFAs, adding a distinct layer of cerebrovascular protection.

#### 3.1.4 Brain Vascular Protection by Myo‐Inositol

Myo‐inositol, another exercise‐derived metabolite, contributes to cerebrovascular protection by enhancing mitochondrial function and ATP production, thereby reducing vascular fragility and risk of rupture. Moderate exercise‐induced ROS also activate endogenous antioxidant defenses, but excessive ROS may drive NF‐κB–mediated inflammation and vascular injury (Gomez‐Cabrera et al. 2008; Chen et al. 2018). In this context, TPPs, and especially EGCG, can work in synergy by scavenging free radical species, stabilizing mitochondria, and inhibiting matrix metalloproteinases, thereby helping to preserve vascular function (Tipoe et al. 2007). Collectively, lactate, SCFAs, BHB, and myo‐inositol extend the protective effects of exercise beyond energy metabolism, conferring systemic vascular and neuroprotective benefits. Their actions converge with TPPs to strengthen mitochondrial function, redox homeostasis, and cerebrovascular integrity (Figure 2) (Nobari et al. 2021).

### 3.2 Mechanisms of Exercise Metabolites in Brain Vascular Protection

#### 3.2.1 Anti‐Inflammatory and Antioxidant Mechanisms of Exercise Metabolites

Exercise inevitably elevates the production of ROS. While moderate exercise activates adaptive antioxidant defenses by upregulating GPx and glutathione reductase (GR), thereby reducing inflammation, excessive or prolonged intensity can disrupt redox balance and lead to tissue injury (Done and Traustadóttir 2016). ROS function as signaling molecules regulating growth and apoptosis, but uncontrolled accumulation damages proteins, lipids, and DNA, driving cancer, cardiovascular, and neurodegenerative diseases (Kruk et al. 2019). Diet also modulates redox responses. The Mediterranean diet, enriched in MUFAs, polyphenols, and n‐3 PUFAs, improves insulin sensitivity, suppresses cytokines, reduces adipose oxidative stress, and increases SCFA production, thereby reinforcing gut–brain axis function (Haro et al. 2016). Exercise and TPPs act synergistically to maintain redox balance. Both activate the Nrf2/ARE pathway and suppress NF‐κB, enhancing antioxidant enzyme activity (SOD, GPx), stabilizing endothelial function, and reducing the risk of related diseases (Figure 4). Tea and its active components, such as catechins, anthocyanins, and polyphenols, can enhance mitochondrial efficiency. Meanwhile, the metabolic products generated by exercise (such as lactic acid and short‐chain fatty acids) can inhibit TNF‐α and IL‐6, thereby jointly reducing brain vascular damage (Haghighatdoost and Hariri 2019; Rojano‐Ortega 2021; Liu et al. 2025). The hormetic interaction between ROS and nitric oxide (NO) in the endothelium may explain a wider range of benefits. Intermediate concentrations of ROS enhance the bioavailability of NO and promote vascular adaptation, whilst high concentrations of ROS can generate peroxynitrite and eliminate NO's hormetic effects or its further downstream protective effects. Regular moderate exercise thus reduces the incidence of ROS‐related disorders, including cardiovascular diseases, stroke, Alzheimer's disease, diabetes, and certain types of cancer (Campbell et al. 2019; Quan et al. 2020; Liu et al. 2024).

#### 3.2.2 The Vascular Stabilizing Effects of Exercise Metabolites

Chronic low‐grade inflammation underlies many cardiovascular diseases, including atherosclerosis, NAFLD, hypertension, and type 2 diabetes (Cifuentes et al. 2025). Dysregulated interactions between metabolic tissues and immune cells, exacerbated by gut dysbiosis, further promote systemic inflammation through PRRs recognizing LPS and other pathogen‐associated molecules (Belizário et al. 2018). Among microbial metabolites, trimethylamine N‐oxide (TMAO) has emerged as a pathogenic mediator of vascular injury. By activating NF‐κB, inflammasomes, and oxidative stress, TMAO accelerates atherosclerosis and increases stroke and CAD risk. Elevated TMAO levels correlate strongly with adverse cardiovascular outcomes, making it a biomarker of vascular risk. Similarly, phenylacetylglutamine (PAGln) promotes adrenergic receptor–mediated thrombogenesis, serving as both a diagnostic marker and predictor of β‐blocker responsiveness (Krishnamoorthy et al. 2024). Protective factors counterbalance these risks. Plant‐derived polyphenols regulate microbiome‐bile acid metabolism, improving lipid homeostasis and suppressing inflammation, thereby reducing diet‐induced cerebrovascular injury (Jiang et al. 2025). Exercise further enhances vascular stability by improving cardiac output, endothelial function, and vascular elasticity, while lowering visceral fat and optimizing lipid profiles. Through antioxidant effects, exercise reduces vascular oxidative stress, preserves endothelial integrity, and promotes vasodilation. Notably, exercise remodels the gut microbiome, enriching *Bifidobacterium* and *Lactobacillus* while reducing pro‐inflammatory species. This promotes SCFA production, particularly butyrate, which strengthens the intestinal barrier, attenuates inflammation, and prevents cerebrovascular lesions. Circulating miR‐210 has also been identified as a biomarker of cerebrovascular oxidative stress, offering diagnostic value for monitoring exercise‐mediated vascular protection (Bei et al. 2024). Together, exercise metabolites enhance vascular resilience by regulating oxidative stress, immune responses, and microbiota composition. These effects not only protect against cerebrovascular injury but also complement the actions of TPPs, laying the groundwork for synergistic neurovascular protection discussed in the following section. Although exercise metabolites are rarely examined in ICH–specific models, convergent evidence supports their relevance to hemorrhagic vulnerability through shared neurovascular mechanisms. Exercise‐derived metabolites enhance endothelial and mitochondrial function, suppress inflammation, and preserve blood–brain barrier integrity, thereby indirectly stabilizing cerebral small vessels and potentially reducing hemorrhagic vulnerability.

## 4 Mechanistic Basis of Tea Polyphenols and Exercise in Brain Vascular Protection

The synergy between TPPs and exercise is framed within an integrated perspective encompassing mechanistic, phenotypic, and temporal dimensions. Mechanistically, both interventions converge on key neurovascular pathways, including Nrf2–ARE–mediated antioxidant defense, suppression of NF‐κB–driven inflammation, enhancement of eNOS/NO bioavailability, preservation of mitochondrial function, maintenance of tight‐junction integrity, and stabilization of the microvasculature. Although exercise and TPPs act through distinct upstream stimuli, their coordinated regulation of these shared targets supports endothelial function, blood–brain barrier integrity, and cerebral perfusion. At the phenotypic level, combined interventions consistently demonstrate greater improvements than either intervention alone in oxidative stress and inflammatory markers, endothelial‐dependent vasodilation, metabolic regulation, blood–brain barrier integrity, and resistance to microvascular injury, particularly under conditions of metabolic stress and aging. In addition, a temporal dimension of synergy is evident, whereby regular exercise establishes an adaptive physiological and microbial milieu that enhances the efficacy and durability of polyphenol‐mediated effects, while sustained polyphenol intake facilitates recovery and adaptive remodeling following exercise‐induced oxidative or inflammatory challenges. Together, this integrated framework provides a coherent basis for interpreting the synergistic neurovascular benefits of TPPs and exercise discussed throughout this chapter.

### 4.1 Synergistic Mechanisms of Tea Polyphenols and Exercise in Neurovascular Protection

#### 4.1.1 Synergistic Regulation of Oxidative Stress by Tea Polyphenols and Exercise

**FIGURE 5:**
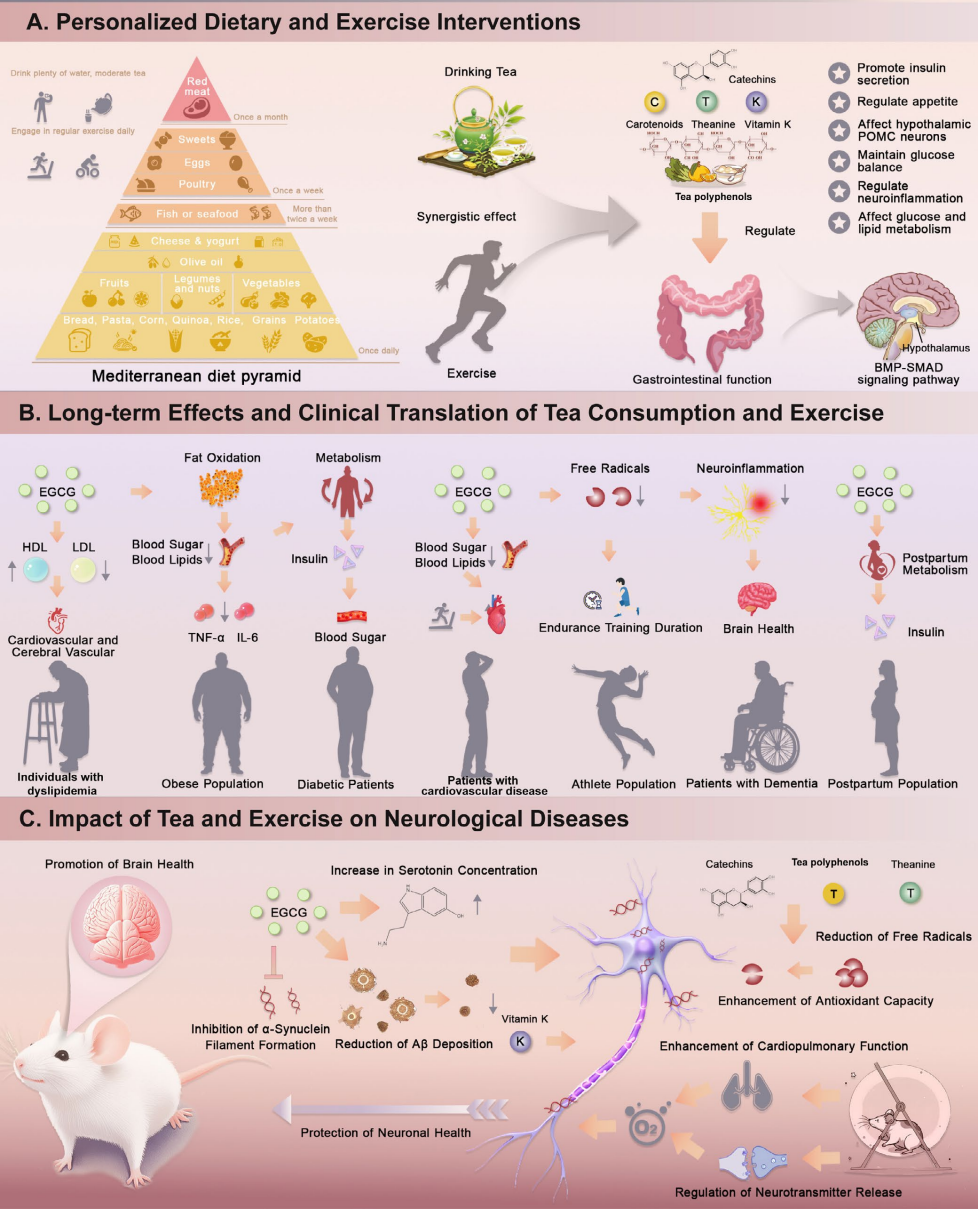
Synergistic effects of tea consumption and exercise on metabolic health, neurological diseases, and long‐term health outcomes. (A) Personalized dietary and exercise interventions: This panel outlines the synergistic effects of drinking tea and engaging in exercise on various health outcomes. The Mediterranean diet pyramid highlights the importance of a balanced diet, with an emphasis on tea rich in catechins, theanine, and other polyphenols, and regular physical exercise. These lifestyle factors improve insulin sensitivity, regulate appetite, maintain glucose and lipid metabolism, and modulate neuroinflammation, benefiting overall gastrointestinal and brain health. (B) Long‐term effects and clinical translation: This panel details the effects of tea consumption, particularly EGCG, and exercise on metabolic processes and health conditions. It shows how EGCG influences fat oxidation, metabolism, and blood sugar levels, while regulating inflammatory markers like TNF‐α and IL‐6. The figure also demonstrates how these interventions benefit specific populations, such as those with obesity, diabetes, cardiovascular diseases, and dementia, as well as improving exercise endurance and brain health. Postpartum metabolism is also addressed, with a focus on the positive effects on insulin regulation. (C) Impact of tea and exercise on neurological diseases: This section highlights the neuroprotective benefits of tea and exercise. It shows how tea compounds, including EGCG, other catechins, and theanine, reduce free radicals, enhance antioxidant capacity, and promote brain health by inhibiting α‐synuclein filament formation and reducing amyloid β (Aβ) deposition. Additionally, these compounds help regulate neurotransmitter release and protect neuronal health, offering potential supportive benefits for neurological diseases such as Parkinson's and Alzheimer's.

Controlled ROS production and inflammatory responses are fundamental protective mechanisms, eliminating pathogens and initiating tissue repair (Weavers et al. 2019). However, excessive or persistent ROS production disrupts cellular homeostasis, damages proteins, lipids, and DNA, and promotes chronic inflammation and metabolic dysfunction (Fan et al. 2019; Milkovic et al. 2019). Under pathological conditions, such as hyperglycemia, ROS accumulation further impairs endogenous antioxidant defenses (Sun et al. 2020). Nrf2 serves as a master regulator of redox homeostasis. Moderate activation of Nrf2 enhances antioxidant capacity, whereas overwhelming stress may compromise adaptive responses (Dai et al. 2024). Exercise and polyphenolic compounds activate Nrf2 via distinct yet convergent molecular triggers. Exercise‐induced transient ROS and redox‐active TPPs can modulate cysteine residues on KEAP1, facilitating Nrf2 dissociation and transcriptional activation of antioxidant genes (Andrés et al. 2024). Importantly, phenotypic evidence from both animal and human studies supports the notion that combined interventions can exceed the effects of single‐component interventions. In rats, treadmill running combined with curcumin supplementation significantly upregulated Nrf2 protein levels beyond either intervention alone (Sahin et al. 2021). In humans, polyphenol‐rich tart cherry juice consumed during exercise modulated oxidative stress markers, suggesting potential translational relevance. Plant‐derived polyphenols, including tea polyphenols and extracts from Rutaceae fruits, enhance endogenous antioxidant defenses, mitigating exercise‐induced oxidative injury. Rodent studies show increased serum SOD activity and decreased LDH levels following tea polyphenol supplementation during exhaustive exercise (Bi et al. 2023; Berilli et al. 2022; Yang et al. 2020). Moderate exercise itself promotes cardiovascular and neuromuscular adaptation and upregulates antioxidant systems, whereas high‐intensity or unaccustomed exercise may induce mitochondrial stress, membrane damage, and excessive ROS production (Da Rocha et al. 2019; Stožer et al. 2020). Polyphenols counteract these effects by limiting oxidative damage and supporting post‐exercise recovery (D'Angelo 2019; Gonçalves et al. 2022). Combined interventions of exercise with polyphenols further reinforce antioxidant defenses. GTE supplementation alongside exercise reduces hepatic lipid accumulation and promotes mitochondrial biogenesis in non‐alcoholic fatty liver disease models (Sun et al. 2022). Catechins in GTE, including EGCG, ECG, CG, and GCG, exhibit cooperative antioxidant activity (Colon and Nerin 2012). Co‐administration of EGCG with curcumin, vitamin C, quercetin, folic acid, or Osmanthus extracts enhances absorption and metabolic regulation (Pandit et al. 2019; Xie et al. 2022; Mao et al. 2017). These multi‐component interventions synergistically enhance enzymatic (SOD, CAT, GPx) and non‐enzymatic (vitamins C and E, glutathione) antioxidant defenses via the Nrf2–ARE pathway, promoting lipid oxidation, energy utilization, and protection against metabolic syndrome (Malaguti et al. 2013; Esmaeilpanah et al. 2021). Long‐term integration of tea polyphenol intake with regular exercise further illustrates temporal synergy, whereby exercise‐induced adaptive priming enhances the durability of polyphenol‐mediated antioxidant responses, contributing to improved mitochondrial function, endurance capacity, and healthy aging (Figure 5) (Derbyshire et al. 2021; Hoseini et al. 2016).

#### 4.1.2 Synergistic Anti‐Inflammatory Effects of Tea Polyphenols and Exercise

Clinical studies provide phenotypic evidence of synergy, as combined interventions reduce inflammatory markers to a greater extent than exercise alone. This effect is partly mediated by convergent suppression of NF‐κB signaling. Oxidative stress impairs endothelial NO bioavailability and exacerbates vascular inflammation, whereas combined tea polyphenol intake and exercise reduce ROS generation, inhibit NF‐κB activation, and downregulate pro‐inflammatory mediators such as IL‐1β, TNF‐α, and COX‐2 (Hussain et al. 2016). Compared with TPS or pigments, total TPPs exhibit stronger anti‐inflammatory efficacy, protecting mitochondrial integrity and preventing ROS‐induced apoptosis via modulation of Bcl‐2 and caspase‐3 pathways (Wei et al. 2023). Novel enzyme‐treated green tea extracts (YLGT) additionally modulate gut microbiota, enhance SCFA production, activate FFAR2/3, and suppress TLR4/NF‐κB signaling, restoring gut barrier integrity (Tian et al. 2024). EGCG inhibits intestinal and systemic inflammation by suppressing NF‐κB, MAPK, and NLRP3 inflammasome activation, reducing cytokine release, and preserving tight junction integrity (Byun et al. 2018). Polyphenols from lychee, thyme, and Pu‐erh tea similarly inhibit TLR4‐mediated NF‐κB signaling and promote SCFA‐mediated epithelial repair, while also modulating the gut–brain axis to attenuate blood–brain barrier (BBB) disruption and neuroinflammation (Hu et al. 2023). In addition, exercise‐derived metabolites such as lactate contribute to temporal synergy by maintaining endothelial and BBB integrity during inflammatory stress, thereby prolonging and amplifying the anti‐inflammatory effects of TPPs (Liu et al. 2017). Exercise combined with polyphenols or other bioactive extracts, such as aloe or matcha, may enhance NK cell activity, reduce IL‐1β, IL‐6, and TNF‐α, and improve glucose and lipid metabolism more effectively than exercise alone (Serrano et al. 2018). Collectively, these findings demonstrate that TPPs and exercise synergistically suppress inflammatory signaling, limit adipose tissue inflammation, and improve metabolic homeostasis (Farhan 2023; Joo et al. 2012).

#### 4.1.3 Synergistic Modulation of the Gut‐Brain Axis by Tea Polyphenols and Exercise

The gut microbiota plays a pivotal role in brain health, and its dysregulation has been implicated in neurodevelopmental and neurodegenerative disorders (Yahya Alkinani and Westwood Clini 2024). For instance, *Klebsiella* overgrowth in preterm infants predicts brain injury and elicits pro‐inflammatory immune responses. Exercise positively influences microbial composition and function (Zheng et al. 2025). Specifically, aerobic training enhances the abundance of *Bifidobacterium* and improves microbial diversity, whereas resistance training enriches butyrate‐producing taxa, such as *Faecalibacterium* and *Lachnospiraceae*, thereby increasing SCFA production (Barzak et al. 2022). These adaptations support intestinal barrier integrity, anti‐inflammatory signaling, and energy metabolism. TPPs, particularly EGCG, synergize with exercise by selectively promoting SCFA‐producing bacteria, including *Akkermansia*, *Lactobacillus*, and *Bifidobacterium*, thereby reinforcing tight junctions and maintaining barrier function (Truong and Jeong 2022; Tang 2023). Polyphenol‐rich teas, such as green, black, and enzyme‐treated varieties, counteract dysbiosis induced by high‐fat diets or colitis, suppressing pathogenic genera like *Desulfovibrio*, *Escherichia*, and *Clostridium* while enriching beneficial taxa such as *A. muciniphila* , *Allobaculum*, and *Muribaculaceae* (Xu 2024; Gao et al. 2020). Mechanistically, SCFAs serve as enterocyte energy substrates, inhibit histone deacetylases, and modulate TLR4/NF‐κB signaling, thereby reducing pro‐inflammatory cytokine production (Tang 2023). The gut–brain axis represents a convergent biological interface through which TPPs and exercise exert synergistic effects. Exercise primarily enhances microbial diversity and SCFA availability, whereas TPPs selectively enrich SCFA‐producing taxa and suppress pathobionts. Together, these interventions generate phenotypic synergy, reflected by improved intestinal barrier integrity, reduced systemic inflammation, and preserved BBB function (Parada Venegas et al. 2019; Xu 2024; Seki et al. 2021). These outcomes exceed those typically observed with either intervention alone. SCFAs and polyphenol metabolites may influence the brain directly or indirectly through blood–brain barrier–related and systemic signaling mechanisms, mitigating neuroinflammation, preserving neuronal function, and supporting cognitive processes such as memory, attention, and learning. In parallel, TPPs directly inhibit TLR4/NF‐κB and MAPK pathways while activating Nrf2/ARE, enhancing antioxidant defenses (Parada Venegas et al. 2019; Kim et al. 2014). Beyond neuroprotection, the combined intervention improves lipid and glucose metabolism, promotes M2 macrophage polarization, and reduces oxidative stress, collectively supporting systemic and gut–brain health (Hong et al. 2022). Taken together, these findings highlight the gut–brain axis as a convergent target of dietary and lifestyle interventions, underscoring the therapeutic potential of integrating tea consumption with regular exercise to optimize cognitive resilience and metabolic homeostasis (Stevens and Maier 2016; Jayatunga et al. 2022).

### 4.2 Synergistic Improvements in Cerebral Blood Flow and Vascular Function

TPPs and exercise metabolites exert mechanistically convergent yet functionally complementary effects on cerebral blood flow and vascular function (Table 3). TPPs reduce platelet aggregation, promote endothelial repair, and enhance vascular elasticity, thereby increasing resistance to hemodynamic stress. Concurrently, exercise‐derived metabolites such as lactate activate endothelial nitric oxide synthase (eNOS), elevating NO production and promoting vasodilation (Siamwala et al. 2013). Notably, combined interventions outperform single treatments by improving vascular barrier integrity, reducing ischemic injury, and potentially reducing microvascular fragility and hemorrhagic vulnerability. Machine learning–based analyses further support the association with these cardiovascular benefits, including enhanced lipid regulation, vascular remodeling, and cardiac performance. Aging and hypoxia exacerbate vascular dysfunction through elevated oxidative stress and impaired repair mechanisms. In this context, TPPs, particularly EGCG, restore endothelial function by enhancing NO synthesis, reducing MDA accumulation, and activating antioxidant enzymes such as SOD and GPx (Arabzadeh et al. 2022). Exercise complements these effects by improving cardiac output, modulating the Bax/Bcl‐2 ratio, and attenuating cardiomyocyte apoptosis. Animal studies show that aerobic training down‐regulates HIF‐1α via FIH‐1 and sirtuin activation, thereby alleviating hypoxia‐induced cardiac injury. When combined, tea and exercise synergistically reduce caspase‐3 and Bax expression, increase Bcl‐2 levels, and strengthen vascular and myocardial resilience against oxidative stress and apoptosis. Beyond vascular protection, TPPs and exercise jointly improve metabolic health. EGCG enhances insulin sensitivity, suppresses hepatic fat accumulation, and inhibits adipogenesis, whereas exercise promotes fatty acid oxidation and glucose utilization. Their combination markedly lowers fasting glucose, insulin, and lipid levels, emphasizing a protective role against metabolic syndrome (Sae‐Tan et al. 2014). In metabolic disease models, EGCG reduces systemic inflammation via TNF‐α and IL‐6 suppression, while exercise metabolites activate AMPK signaling to limit visceral fat accumulation. The MMP‐9/TIMP‐1 axis has been identified as a potential molecular mediator of these effects, and certain traditional Chinese medicine formulas, such as Si Miao Wan, demonstrate comparable synergy with exercise by enhancing SCFA production and lowering blood pressure (Jin et al. 2023). In conclusion, TPPs and exercise synergistically enhance vascular function, mitigate oxidative stress, and improve metabolic homeostasis. Their combined effects on endothelial repair, redox balance, and inflammatory regulation may help reduce vulnerability to stroke, atherosclerosis, and cerebral hemorrhage, while also alleviating systemic metabolic dysfunctions such as NAFLD. This integrated strategy represents a promising avenue for cardiovascular protection and the prevention of cerebrovascular complications (Liu, De Bruijn, et al. 2020; Edward and Cornwell 2022). Although direct evidence in ICH models remains limited, the convergence of effects on BBB integrity, endothelial repair, redox balance, and inflammatory regulation supports a plausible synergistic protective role against cerebrovascular fragility and spontaneous ICH.

**TABLE 3:** Core integrated mechanistic framework of tea polyphenols and exercise in neurovascular protection.

Mechanistic domain	Key pathways/targets	Synergistic actions of tea and exercise	System‐level outcomes
Antioxidant & anti‐inflammatory regulation	Nrf2/Keap1, TLR4/MyD88/NF‐κB	Moderate exercise‐induced ROS promotes Nrf2 nuclear translocation, while EGCG enhances antioxidant enzyme activity; jointly suppress TLR4/MyD88/NF‐κB signaling and may promote anti‐inflammatory cytokine responses	Reduced oxidative stress and systemic/neuroinflammation
Gut microbiota–metabolite axis	SCFAs, GPR41/43, ZO‐1/occludin/claudin‐1	Tea polyphenols promotes the growth of beneficial taxa such as *Akkermansia* and *Bifidobacterium*; exercise increases SCFA production and reduces LPS translocation	Enhanced gut barrier integrity and immune homeostasis
Vascular structure and function	eNOS/NO/cGMP, MMP‐9‐related extracellular matrix remodeling	EGCG preserves endothelial mitochondrial function, while exercise enhances shear stress–induced eNOS phosphorylation; jointly inhibit MMP‐9 activity	Improved cerebrovascular perfusion and vascular stability
Metabolic reprogramming	AMPK/SIRT1, PGC‐1α, mitochondrial biogenesis	Exercise activates AMPK‐driven fatty acid oxidation; EGCG may improve insulin sensitivity and support mitochondrial homeostasis	Improved glucose–lipid homeostasis
Neurocognitive protection	BDNF, BBB integrity, neuroinflammatory signaling	Tea polyphenols upregulate neurotrophic factors, while exercise enhances cerebral blood flow and suppresses BBB inflammation	Cognitive resilience and reduced neuronal apoptosis
Microbiota‐immune interaction	ZO‐1, occludin, claudin‐1, LPS	Tea polysaccharides may strengthen epithelial tight junctions, while exercise may limit endotoxin translocation	Strengthened mucosal immune barrier

*Note:* Tea polyphenols, particularly EGCG, and exercise may exert coordinated protective effects on systemic and cerebrovascular health through coordinated regulation of antioxidant defense, inflammation, gut microbiota, vascular function, metabolism, and cognitive resilience.

Abbreviations: AMPK, AMP‐activated protein kinase; BBB, blood–brain barrier; BDNF, brain‐derived neurotrophic factor; DPP4, dipeptidyl peptidase‐4; EGCG, epigallocatechin gallate; eNOS, endothelial nitric oxide synthase; GPR41/43, G protein–coupled receptors 41/43; IL, interleukin; KEAP1, Kelch‐like ECH‐associated protein 1; LPS, lipopolysaccharide; MMP‐9, matrix metalloproteinase‐9; NF‐κB, nuclear factor kappa B; NO, nitric oxide; NRF2, nuclear factor erythroid 2–related factor 2; PGC‐1α, peroxisome proliferator‐activated receptor gamma coactivator 1‐alpha; ROS, reactive oxygen species; SCFAs, short‐chain fatty acids; SIRT1, sirtuin 1; TLR4, Toll‐like receptor 4; ZO‐1, zonula occludens‐1.

## 5 Potential Mechanism Model

### 5.1 Regulation of Gut Microbiota and Metabolites by Tea Consumption and Exercise

The gut microbiota represents a central upstream regulatory hub through which tea consumption and exercise exert coordinated neurovascular effects. Rather than acting through isolated biological pathways, these lifestyle interventions reshape microbial ecology and metabolic output in a complementary manner, thereby influencing systemic inflammation, metabolic homeostasis, and brain vascular health (Table 3) (Yang et al. 2024). TPPs predominantly exert selective pressure on microbial composition and intestinal barrier integrity, whereas exercise primarily enhances microbial diversity and metabolic activity. Together, these effects converge to optimize the production of gut‐derived metabolites, particularly SCFAs, while limiting endotoxin translocation. The coordinated remodeling of gut microbiota contributes to reduced systemic inflammatory tone, improved lipid and glucose metabolism, and enhanced energy utilization. Over time, combined tea consumption and regular exercise synergistically decrease abdominal adiposity, improve lipid profiles, and enhance skeletal muscle endurance (Angelina Faraldo Corrêa et al. 2021; Liu, Luo, et al. 2020). Beyond metabolic regulation, gut microbiota‐derived signals also support the gut–brain axis by modulating immune, endocrine, and neural communication. This integrated microbial–metabolic framework provides a foundational mechanism linking dietary and physical activity interventions to sustained neurovascular resilience (Wu et al. 2021; Zhang et al. 2022).

### 5.2 Activation of Antioxidant and Anti‐Inflammatory Pathways by Tea and Exercise

Oxidative stress and inflammation constitute shared pathological drivers underlying neurovascular dysfunction. TPPs and exercise converge on these processes by targeting interconnected redox‐ and inflammation‐sensitive signaling networks. Rather than duplicating individual molecular pathways, their combined actions reinforce endogenous antioxidant defenses while promoting the resolution of chronic low‐grade inflammation (Tu et al. 2019). Tea‐derived bioactive compounds contribute to redox balance by scavenging reactive species and supporting cellular antioxidant capacity, whereas exercise‐induced redox signaling enhances adaptive stress responses and metabolic efficiency. When integrated, these interventions amplify endogenous defense systems, attenuate lipid peroxidation, and stabilize cellular homeostasis across multiple organs (Ellinger et al. 2011). Importantly, this convergence also extends to metabolic tissues, where coordinated regulation of oxidative stress and inflammation supports hepatic lipid handling, insulin sensitivity, and mitochondrial function (Hodges et al. 2020). Collectively, these effects establish a systemic environment that is resistant to oxidative injury and inflammatory overload, thereby supporting long‐term neurovascular protection.

### 5.3 Stabilization of Vascular Structure and Function Through Tea and Exercise

From a vascular systems perspective, tea consumption and exercise jointly contribute to the stabilization of endothelial structure and function. This integrated regulation preserves vascular elasticity, limits maladaptive remodeling, and enhances resistance to hemodynamic and metabolic stressors. TPPs support endothelial integrity and vascular adaptability, while exercise improves cardiac output, vascular responsiveness, and lipid handling (Larson et al. 2006; Oliviero et al. 2013). The synergistic effects of these interventions may reduce susceptibility to vascular calcification, atherosclerosis, and microvascular injury. By maintaining redox balance and inflammatory control within the vascular microenvironment, combined tea and exercise interventions promote long‐term vascular resilience. This structural stabilization may help lower cardiovascular risk and protect cerebral microcirculation, thereby potentially reducing the likelihood of cerebrovascular events and age‐related vascular decline (Cheng et al. 2017; Du et al. 2023; Vossen et al. 2019).

### 5.4 Cognitive Enhancements Through Tea and Exercise

At the functional outcome level, the combined effects of tea consumption and regular exercise extend beyond vascular protection to encompass cognitive resilience and brain health. Through integrated regulation of vascular function, metabolic homeostasis, and inflammatory balance, this lifestyle‐based strategy supports sustained cerebral perfusion and neuronal integrity (Tran et al. 2023; Mancini et al. 2017). Over the long term, these adaptations contribute to improved cognitive performance, particularly in domains sensitive to aging and metabolic stress. By reinforcing neurovascular coupling and preserving the integrity of brain‐supporting systems, the combination of tea intake and exercise may represent a promising non‐pharmacological approach to delaying cognitive decline and reducing vulnerability to neurodegenerative processes (Feng et al. 2012; Yu et al. 2024). Collectively, this integrative mechanistic model highlights how coordinated dietary and physical activity interventions can promote brain vascular health, cognitive longevity, and overall metabolic stability. The combined effects of TPPs and exercise on gut microbiota, oxidative stress, and inflammation have important implications for reducing hemorrhagic vulnerability. Both interventions synergistically preserve blood–brain barrier (BBB) integrity, enhance endothelial function, and regulate redox homeostasis, all of which are critical for maintaining cerebral small vessel stability and potentially reducing susceptibility to spontaneous ICH. Furthermore, the gut–brain axis and metabolic regulation facilitated by tea and exercise contribute to long‐term neurovascular resilience, which may help reduce vulnerability to cerebrovascular events, including hemorrhagic stroke. By stabilizing vascular structure and function, these interventions may reduce vascular fragility and provide a plausible protective framework against ICH.

## 6 Clinical Translation and Future Perspectives of Tea and Exercise Interventions

With the advancement of personalized and preventive medicine, integrating tea consumption with structured exercise programs represents a promising clinically translatable, non‐pharmacological strategy for the prevention and management of cerebrovascular diseases. From a translational perspective, individualized interventions based on metabolic profiles, lifestyle patterns, genetic background, and disease risk stratification may maximize therapeutic efficacy while minimizing adverse effects. Longitudinal clinical trials and real‐world cohort studies are essential to validate mechanistic findings derived from preclinical models, particularly regarding stroke rehabilitation, vascular repair, metabolic regulation, and neuroprotection. Such evidence will facilitate the translation of tea–exercise synergy into clinical guidelines and public health strategies. In addition, the development of functional foods or supplements enriched with TPPs and exercise‐induced metabolites may further enhance clinical applicability in high‐risk populations (González‐Sarrías et al. 2017).

### 6.1 Translational Potential of Personalized Dietary and Exercise Interventions

Personalized dietary and exercise interventions have substantial translational value across diverse clinical populations, including older adults, individuals with obesity, athletes, and patients with metabolic or inflammatory disorders. Clinically applicable dietary patterns, such as the Mediterranean diet (MD) and low‐FODMAP strategies, have demonstrated feasibility in optimizing gut health while reducing exercise‐induced gastrointestinal symptoms (Parnell et al. 2020; Lis et al. 2016). The integration of nutritional supplementation with genomics, metabolomics, and microbiomics enables precision health strategies that can be implemented in clinical and preventive settings (Lagoumintzis and Patrinos 2023). Adjusting green tea intake, polyphenol dosage, and exercise modality or intensity allows for targeted modulation of lipid metabolism, antioxidant defense, inflammatory pathways, and gut–brain axis signaling (Yan et al. 2020). Matcha, a clinically relevant form of green tea powder rich in catechins, has shown benefits in improving insulin resistance, serum antioxidant enzymes, lipid metabolism, and hypothalamic BMP‐SMAD signaling, particularly when combined with exercise (Zhou et al. 2024). Regular intake is associated with improvements in neurological, cardiovascular, and metabolic functions, supporting its potential inclusion in dietary recommendations for metabolic and cerebrovascular risk management. Catechins and theanine further alleviate fatigue and psychological stress, supporting metabolic recovery, while vitamins (C, D, E) and minerals (magnesium, zinc, selenium) synergistically protect telomere integrity, DNA stability, redox balance, and vascular health (Hoseini et al. 2016; Cai et al. 2023). MUFAs and polyphenols in extra virgin olive oil, core components of the MD, enhance endothelial function, reduce LDL oxidation, improve gut microbiota, and promote SCFA production, thereby potentially contributing to cerebrovascular risk reduction (Raparthi et al. 2022). Genetic polymorphisms and metabolic phenotypes highlight the necessity of personalized clinical protocols rather than uniform recommendations. Dietary antioxidants, encompassing polyphenols, vitamins, and minerals, play a significant role in mitigating inflammation and oxidative stress. Concurrently, physical activity, which includes both occupational and recreational forms, contributes to maintaining a balance between pro‐inflammatory and anti‐inflammatory processes, thereby enhancing cardiovascular, metabolic, cognitive, and mental health (Holtermann et al. 2018). Given the limited stability and bioavailability of tea bioactives such as EGCG and TFs, advanced delivery systems and stabilized catechin derivatives represent important translational innovations to enhance clinical efficacy (Bakun et al. 2023).

### 6.2 Clinical Evidence and Long‐Term Translational Outcomes

#### 6.2.1 Long‐Term Clinical Effects Across Different Populations

Accumulating clinical and translational evidence supports the long‐term protective effects of combined tea consumption and exercise across multiple populations. GTE promotes glutathione biosynthesis, enhancing endogenous antioxidant capacity in individuals with metabolic syndrome (Blumberg et al. 2015). While GTE does not markedly reduce cumulative exercise‐induced muscle damage, it preserves neuromuscular performance and mitigates oxidative stress in untrained males (Bowtell and Kelly 2019). Catechins improve obesity, glucose and lipid metabolism, inflammation, and gut microbiota (Ohishi et al. 2016). Combining green tea with exercise enhances cardiovascular function, cardiac output, vascular endothelial health, and elasticity, reducing hypertension and atherosclerotic risk. Studies suggest that older adults who undergo these combined interventions may show improvements in cognitive domains, including memory and learning (Walker et al. 2015). In obese and diabetic populations, TPPs with exercise increase fat oxidation, regulate lipid metabolism, decrease visceral fat, lower TNF‐α and IL‐6, enhance insulin sensitivity, protect pancreatic β‐cells, and support long‐term glycemic control (Langlois et al. 2021). In patients with cardiovascular diseases, green TPPs reduce blood pressure and lipid levels, while exercise improves cardiac output and vascular adaptability, collectively lowering cardiovascular risk (Figure 5) (Nystoriak and Bhatnagar 2018). In athletes, polyphenols mitigate exercise‐induced oxidative stress, supporting recovery and endurance, while exercise optimizes performance and cardiovascular health (Kuo et al. 2015). Elderly individuals with dementia and pregnant/postpartum women benefit from neuroprotective, anti‐inflammatory, and metabolic effects of combined interventions. Animal studies indicate green tea alleviates tissue damage and cardiovascular risk under environmental stress, hypoxia, and intense exercise, partly via antioxidant and vasodilatory mechanisms (Jówko et al. 2015). Pu‐erh tea, rich in antioxidants and microbiota‐modulating compounds, affects tryptophan metabolism, serotonin levels, and neuroprotection, potentially helping to alleviate depressive symptoms (Savitz 2016). EGCG reduces hepatic lipid accumulation and improves serum lipid profiles through HIF1α and LDLR regulation, consistent with PCSK9‐targeted mechanisms (Singh et al. 2011). High‐fat diets impair BBB integrity, leptin signaling, and hypothalamic energy regulation (Timper and Brüning 2017). Matcha combined with voluntary exercise improves insulin resistance, glucose metabolism, serum CAT, NAD‐related pathways, lipid metabolism, and hypothalamic BMP‐SMAD signaling, demonstrating systemic and central regulatory potential (Zhou et al. 2024, 2020). Theanine supports central and peripheral nervous system recovery, muscle adaptation, protein synthesis, and resistance training outcomes (Kochman et al. 2020; Kika et al. 2024; Unno et al. 2018; Shigeta et al. 2023). Fermented beverages such as kombucha provide beneficial microbes and bioactives, counteracting diet‐induced gut dysbiosis and inflammation (Costa et al. 2022). Endurance training with catechin supplementation improves mitochondrial function and muscle strength in older adults and sarcopenic populations (Haramizu et al. 2011). These findings collectively indicate that tea–exercise interventions have broad translational relevance across preventive, rehabilitative, and performance‐oriented clinical settings.

#### 6.2.2 Development of Clinically Functional Products

Functional foods and beverages containing TPPs, exercise‐derived metabolites, probiotics, prebiotics, dietary fiber, vitamins, minerals, and amino acids are increasingly used to promote health and prevent disease (Temple 2022). Combining tea and exercise metabolites presents opportunities in translational medicine, with potential to reduce cerebrovascular disease risk in high‐risk populations (Zhang et al. 2016). Catechins enhance mitochondrial function, increase fat oxidation, reduce oxidative stress, and support recovery, particularly in athletes engaged in prolonged aerobic or strength training (Sugita et al. 2016). Polyphenols exert integrated antioxidant, anti‐inflammatory, anti‐mutagenic, and vasodilatory effects, improving immune function, metabolic regulation, muscle strength, aerobic performance, and mitigating exercise‐induced oxidative stress (Ferreira et al. 2016). Functional foods such as matcha, combined with exercise, activate hippocampal signaling, promote insulin secretion, enhance metabolic regulation, modulate BMP‐SMAD pathways implicated in obesity and T2DM, and support CNS‐specific metabolic and neuroprotective roles (Table 4) (Zhou et al. 2024). Dietary modulation also impacts gut microbiota composition. High‐fat, high‐fructose diets increase pathogenic taxa such as *Proteobacteria*, while healthier diets and fermented beverages enhance propionate production and the beneficial genus *Adlercreutzia* (Costa et al. 2022). Functional compounds, including turmeric, soy protein, and ginseng, improve skeletal muscle function. Catechins and oligophenols suppress circulating cytokines, inhibit NF‐κB signaling, activate longevity factors such as sirtuin1, and delay age‐related decline. Long‐term EGCG supplementation extends lifespan in animal models, while inhibition of NF‐κB‐mediated muscle protein degradation reduces sarcopenia (Onishi et al. 2018). Taken together, polyphenols represent promising natural interventions that, when combined with exercise, optimize antioxidant and anti‐inflammatory defenses, metabolic regulation, cardiovascular function, neuroprotection, muscle performance, and gut‐brain axis integrity. Evidence supports their use as functional nutritional strategies for enhancing exercise outcomes, promoting recovery, and reducing the risk of chronic diseases (Deley et al. 2017; D'Angelo 2019).

**TABLE 4:** Translational research directions for tea and exercise synergy.

Research focus	Intervention strategy	Core mechanism	Target population
Personalized metabolic regulation	Genomic/metabolomic‐guided tea‐exercise design	Optimizes AMPK/lipolysis, modulates gut microbiota	Metabolic disorders/Obese populations
Functional food development	Matcha‐based sports supplements	Activates hippocampal signaling, promotes insulin secretion, modulates BMP‐SMAD and metabolic regulation	Athletes/Fitness enthusiasts; metabolic‐risk populations
Neurodegenerative disease intervention	EGCG/tea polyphenols + regular physical activity	Inhibits α‐synuclein aggregation, upregulates BDNF	Neurodegenerative disease patients, especially Parkinson's disease and Alzheimer's disease
Precision cardiovascular protection	Tea polyphenols + Endurance training	Improves endothelial function, vascular elasticity, and eNOS/NO signaling	Hypertensive/Atherosclerosis populations
Gut‐brain axis optimization	Pu‐erh tea + Periodized exercise	Boosts SCFAs production, reshapes gut microbiota	Depression/IBS patients; gut dysbiosis populations
Musculoskeletal health maintenance	Catechin supplementation + Resistance training	Inhibits NF‐κB‐mediated proteolysis and supports mitochondrial function and muscle strength	Sarcopenia/Elderly populations

*Note:* Tea compounds and exercise may synergistically support metabolic, cardiovascular, neurological, gut‐brain, and musculoskeletal health via key signaling and microbiota‐related pathways.

Abbreviations: AMPK, AMP‐activated protein kinase; BDNF, brain‐derived neurotrophic factor; BMP‐SMAD, bone morphogenetic protein‐SMAD signaling pathway; EGCG, epigallocatechin gallate; eNOS, endothelial nitric oxide synthase; IBS, irritable bowel syndrome; NF‐κB, nuclear factor kappa B; NO, nitric oxide; SCFAs, short‐chain fatty acids.

### 6.3 Clinical Implications for Neurological and Cerebrovascular Disorders

Green tea combined with regular exercise exerts potentially synergistic protective effects on cerebrovascular and neurological health. TPPs enhance endothelial function, reduce vascular sclerosis, and decrease platelet aggregation, while exercise improves circulation, hemodynamics, and cardiovascular adaptability, thereby potentially reducing cerebrovascular vulnerability (Green et al. 2017). In line with these vascular benefits, prospective cohort evidence indicates that higher tea consumption is associated with a reduced risk of vascular dementia, highlighting tea intake as a modifiable dietary factor relevant to cerebrovascular‐related cognitive decline (Griffiths et al. 2024). Moderate‐intensity aerobic exercise combined with tea intake enhances cerebrovascular dilation and elasticity, while epigenetic regulators such as miR‐210 further support neuroprotection by modulating myocardial and neural cell proliferation. From a broader aging and neurodegeneration perspective, lifestyle‐based multi‐target strategies integrating physical activity with dietary bioactives have gained increasing attention. Green tea catechin polyphenols, long regarded as dietary antioxidants, are now recognized for their diverse pharmacological activities that may complement exercise‐induced neuroplasticity and stress resilience, thereby contributing to the attenuation of age‐related cognitive decline and neurodegenerative processes (Mandel and Bh Youdim 2012). Among TPPs, EGCG has shown neuroprotective potential in models of Alzheimer's disease, Parkinson's disease, and multiple sclerosis by scavenging ROS, inhibiting lipid peroxidation, modulating blood–brain barrier‐related processes, preserving neuronal morphology, and supporting cognitive function. EGCG also attenuates amyloid‐beta accumulation and increases BDNF expression in vascular dementia and Alzheimer's models (Williams et al. 2016). Notably, emerging clinical evidence suggests that adding EGCG to multimodal lifestyle interventions that include guided physical activity may enhance cognitive outcomes in older adults at elevated risk of cognitive impairment, thereby supporting a potential role for green TPPs as adjuncts to exercise based interventions (Forcano et al. 2025). Consistent with these findings, observational evidence further supports the association between green tea consumption and cognitive health (Zheng et al. 2023). A recent systematic review and meta‐analysis demonstrated that green tea intake is inversely associated with cognitive impairment, including dementia and mild cognitive impairment, across diverse populations, reinforcing the relevance of habitual tea consumption in cognitive health and healthy‐aging strategies (Zhou et al. 2025). Notably, matcha combined with exercise amplifies hippocampal activation, insulin secretion, and metabolic regulation compared with exercise alone (Sae‐Tan et al. 2014). At the molecular level, multiple signaling pathways mediate these effects. EGCG modulates the EGFR/PI3K/Akt/AP‐1 axis to reduce lipid accumulation and protect neurons from oxidative stress, prevents α‐synuclein aggregation, and suppresses pro‐inflammatory cytokine expression in high‐fat diet and insulin‐resistant models. BMP‐SMAD signaling in the CNS regulates appetite, neuroinflammation, and metabolic homeostasis, with BMP7 acting on hypothalamic POMC neurons, BMP6 modulating microglial activity, and Smad3 influencing glucose–lipid metabolism. TPPs may interact with these pathways, though CNS‐specific mechanisms require further investigation. Polyphenol‐rich diets combined with exercise may help mitigate ischemia–reperfusion injury by reducing ROS bursts, enhancing antioxidant enzyme activity, and modulating inflammatory mediators, thereby potentially limiting microthrombi formation and neurological damage (Kassan et al. 2023). Animal studies demonstrate that pre‐ischemia green tea supplementation enhances resilience, while Pu‐erh tea increases BDNF levels and alleviates depression‐like behaviors (Schimidt et al. 2014; Noguchi‐Shinohara et al. 2014). In parallel, physical exercise alone has been extensively documented to reduce the risk of Parkinson's disease and to improve both motor and non‐motor symptoms through mechanisms involving enhanced neurogenesis, synaptogenesis, mitochondrial function, and angiogenesis, providing a complementary biological foundation for combined lifestyle interventions (Xu et al. 2019). Collectively, evidence from mechanistic studies, animal models, observational cohorts, and emerging multimodal intervention trials suggests that tea consumption and physical exercise act on overlapping vascular, metabolic, and neuroplastic pathways relevant to neurodegeneration. Although direct clinical trials specifically designed to test their combined effects remain limited, current findings support the integration of tea‐derived polyphenols and regular physical activity as promising complementary strategies for the prevention and management of cerebrovascular and neurodegenerative disorders (Li et al. 2022).

### 6.4 Translationally Relevant Readouts and Measurable Clinical Endpoints

To enhance the translational relevance of tea–exercise interventions, it is essential to link proposed molecular and physiological mechanisms with clinically accessible and quantifiable endpoints. Several candidate readouts can be incorporated into future clinical trials and cohort studies to validate preclinical findings and mechanistic hypotheses. Cerebrovascular protection attributed to TPPs and exercise can be evaluated using established markers of endothelial and BBB integrity. Circulating biomarkers such as soluble ICAM‐1, VCAM‐1, E‐selectin, and von Willebrand factor reflect endothelial activation and vascular inflammation (Jaime Garcia et al. 2023). Tight‐junction‐associated proteins, including claudin‐5, occludin, and zonula occludens‐1, can be assessed indirectly through circulating extracellular vesicles or cerebrospinal fluid measurements in clinical settings (Sun et al. 2021). Functional vascular assessments, such as flow‐mediated dilation (FMD), pulse wave velocity, and endothelial‐dependent vasodilation tests, provide non‐invasive measures of endothelial function relevant to stroke and small vessel disease risk (Thijssen et al. 2019). Neuroimaging offers robust translational endpoints linking vascular mechanisms to clinical outcomes. Magnetic resonance imaging (MRI) markers, including white matter hyperintensities (WMH), cerebral microbleeds, lacunes, and enlarged perivascular spaces, serve as validated indicators of cerebral small vessel disease progression (Sun and Liu 2025; Voorter et al. 2024). Advanced MRI techniques, such as diffusion tensor imaging and arterial spin labeling, further enable assessment of microstructural integrity and cerebral perfusion changes in response to lifestyle interventions (Jaafar and Alsop 2024; Meoded and Huisman 2019). In addition to imaging indicators, metabolic readouts may also serve as potential translational endpoints. Given that abnormal lipid metabolism is an important risk factor for cardiovascular and cerebrovascular diseases, NMR‐based metabolomic profiling has been shown to predict cardiovascular and all‐cause mortality risk. These metabolomic indicators help to depict vascular risk profiles and provide quantitative tools for risk stratification and for evaluating the long‐term effects of tea consumption and exercise on cardiovascular and cerebrovascular health (Huang et al. 2025). In addition to endogenous metabolic indicators, non‐invasive biomarkers detectable on the body surface also deserve attention. As a non‐invasive surface‐based indicator related to systemic oxidative stress, autofluorescence patterns may serve as potential biomarkers for stroke risk assessment, providing supplementary translational indicators for constructing a multi‐level stroke risk assessment system (Wu et al. 2025). Systemic and central inflammatory responses can be monitored through circulating cytokines, oxidative stress markers, and neurotrophic factors such as BDNF (Grabska‐Kobyłecka et al. 2023). Exercise‐ and polyphenol‐induced neuroplasticity may also be indirectly reflected by cognitive performance metrics and functional imaging readouts (Tari et al. 2025). Overall, integrating biochemical, vascular, and imaging endpoints, molecular and metabolomic markers, as well as emerging non‐invasive biological indicators, provides a feasible translational research framework for evaluating the synergistic effects of tea consumption and exercise on cerebrovascular and neurodegenerative risk reduction.

## 7 Conclusion

Cerebrovascular diseases remain major contributors to morbidity and mortality, highlighting the need for safe and accessible non‐pharmacological strategies. Current evidence suggests that tea polyphenols (TPPs) and exercise‐derived metabolites may support neurovascular resilience through complementary mechanisms, including regulation of oxidative stress, inflammatory signaling, endothelial function, vascular stability, and gut–brain axis homeostasis. When combined, tea consumption and physical exercise may generate additive or synergistic benefits in selected contexts by enhancing endogenous antioxidant capacity, reducing systemic inflammation and neuroinflammation, stabilizing vascular structures, and promoting gut microbiota balance. These coordinated effects may help reduce neurovascular vulnerability and support long‐term cognitive and metabolic health. However, the magnitude and consistency of these benefits vary across studies and populations. Factors such as age, metabolic status, baseline physical activity, tea type, polyphenol composition, dosage, bioavailability, and exercise intensity may influence intervention outcomes. Importantly, direct evidence supporting the prevention of spontaneous intracerebral hemorrhage by tea–exercise interventions remains limited. Therefore, the proposed framework should be interpreted as a biologically plausible and translational model for reducing vascular fragility rather than as a confirmed preventive or therapeutic strategy for ICH. Personalized approaches, including EGCG‐enriched beverages, matcha combined with structured exercise, and polyphenol‐supported dietary patterns, may hold promise for high‐risk populations. Nevertheless, current evidence is still limited by reliance on observational studies, preclinical models, and heterogeneous intervention designs. Future studies should prioritize long‐term randomized controlled trials, standardized characterization of tea preparations and exercise protocols, stratified analyses across diverse populations, and clinically relevant endpoints, including vascular imaging, metabolomic, microbiomic, inflammatory, and cognitive markers. Such research will be essential to clarify the efficacy, safety, and translational applicability of integrating tea consumption with regular physical activity for cerebrovascular and neurological health.

## Author Contributions

**Yazhen Zhang:** conceptualization, investigation, funding acquisition, visualization, validation, methodology, software, formal analysis, project administration, data curation, supervision, resources, writing – original draft. **Yisheng Chen:** writing – review and editing, visualization, validation, investigation, funding acquisition, supervision, resources, conceptualization, methodology. **Qing Yang:** writing – review and editing, validation, visualization, investigation, supervision, software, resources. **Zhaoyuan Huang:** visualization, validation, methodology, conceptualization, software, formal analysis, data curation, resources, investigation, writing – original draft. **Shiwei He:** validation, visualization, formal analysis, data curation, writing – original draft, resources, software. **Chengwan Shen:** data curation, formal analysis, validation, visualization, software, resources, methodology, writing – original draft. **Zijin Sun:** validation, supervision, investigation. **Hua Chen:** validation, visualization, software, formal analysis, data curation, resources, writing – original draft. **Qiangqiang Wang:** methodology, validation, visualization, software, formal analysis, data curation, resources, writing – original draft. **Yuzhen Xu:** validation, investigation, supervision. **John H. Zhang:** validation, writing – review and editing, investigation, supervision. **Guanghui Wu:** writing – review and editing, investigation, validation, visualization, supervision. **Zemin Ou:** supervision, software, investigation, validation, visualization, writing – review and editing. **Zui Zou:** writing – review and editing, validation, investigation, supervision. **Wangzheqi Zhang:** writing – review and editing, validation, investigation, supervision. **Lei Huang:** writing – review and editing, visualization, investigation, supervision. **Shaocong Zhao:** conceptualization, investigation, funding acquisition, visualization, validation, methodology, software, formal analysis, project administration, data curation, resources, supervision, writing – original draft. **Shizhong Zheng:** conceptualization, investigation, methodology, validation, visualization, formal analysis, software, data curation, writing – original draft.

## Funding

This research was supported by the 2025 Fujian Province Education Research Program for Young and Middle‐aged Teachers (Science & Technology, grant number JAT251181), the Fujian Province 2025 Undergraduate University Education Research Program (grant number FBJY20250072), and the National Social Science Fund Project (grant number 23XTY007, Silver Age Sports Science Literacy Promotion Program). Additionally, this project was funded by the Fujian Province Key Social Science Project (grant number FJ2024A016). Moreover, the work received support from the Ningde City 2024 Talent Introduction Research Start‐up Project (grant number 2024Y08) and the Fujian Province Young Scientific and Technological Talent Development Program (grant numbers Min Cai Zhi [2025] 0724 and 25110100011).

## Conflicts of Interest

The authors declare no conflicts of interest.

## Data Availability

This study is a literature‐based review. All data analyzed and presented in this review are sourced from previously published studies, publicly available datasets, and references cited within the article. No new experimental or patient data were generated in this study.
